# One‐Pot Synthesis of Novel *β*‐Carboline‐{α‐Acylaminoamide}‐Bisindole Derivatives: Antibacterial Evaluation, Molecular Docking, and Density Functional Theory Studies

**DOI:** 10.1002/open.202500245

**Published:** 2025-09-17

**Authors:** Ankit Kumar Atri, Lavanya Khullar, Gobind Kumar, Sahil Mishra, Tamanna Dua, Vinay Singh, Kusum Harjai, Parvesh Singh, Vasundhara Singh

**Affiliations:** ^1^ Department of Applied Sciences Punjab Engineering College (Deemed to be University) Sector‐12 Chandigarh 160012 India; ^2^ Department of Microbiology Panjab University Sector‐25 Chandigarh 160014 India; ^3^ School of Chemistry and Physics University of KwaZulu‐Natal P/Bag X54001, Westville Durban 4000 South Africa

**Keywords:** *β*‐carbolines, antibacterial agents, density functional theory studies, molecular hybridization, molecular docking, Ugi reaction

## Abstract

A new series of *β*‐carboline‐{α‐acylaminoamide}‐bisindole hybrids (**12a–l**) is designed and synthesized employing an atom‐economical one‐pot Ugi four‐component reaction (U‐4CR), affording the target compounds in good yields. All synthesized compounds (**12a–l**) are characterized by NMR, infrared, and mass spectrometry and evaluated for their antibacterial activity against both Gram‐positive and Gram‐negative strains. Notably, compounds **12b**, **12g**, and **12h** display comparable minimum inhibitory concentrations values (302–303 µg mL^−1^) against multidrug‐resistant Acinetobacter baumannii and *Pseudomonas aeruginosa*, showing markedly improved activity relative to their parent compounds **6** and **9**, which show weaker inhibition (MIC = 308–623 µg mL^−1^). Molecular docking studies of compounds **12g** and **12h** revealed favorable binding interactions with DNA gyrase, while density functional theory analysis supported their electronic reactivity. These findings highlight the potential of molecular hybridization in the development of novel antibacterial agents.

## Graphical abstract

A new series of *β*‐carboline‐{*α*‐acylaminoamide}‐bisindole hybrids (**12a–l**) employing an atom‐economical one‐pot Ugi four‐component reaction and their antibacterial testing alongside docking studies, is reported.

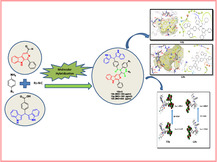

## 1 Introduction

Antimicrobial resistance (AMR) poses a growing and serious threat to global public health.^[^ ^1^ ^]^ It arises when microorganisms, including bacteria, fungi, viruses, and parasites, develop the ability to survive exposure to antimicrobial agents such as antibiotics, antifungals, and antivirals.^[^ ^2^ ^]^ Among these, multi‐ drug‐resistant (MDR)^[^ ^3^ ^]^ bacteria are particularly concerning, as they exhibit resistance to multiple classes of antibiotics, rendering standard treatments ineffective and complicating infection management.^[^ ^4^ ^]^ In response to this escalating threat, the World Health Organization (WHO) released its 2024 Bacterial Priority Pathogens List (BPPL), which identifies 15 families of antibiotic‐resistant bacteria classified into critical, high, and medium priority categories.^[^ ^5^ ^]^ Alarmingly, projections suggest that AMR could surpass cancer as a leading cause of death within the next three decades, with estimates indicating up to 10 million deaths annually by 2050.^[^ ^2^ ^]^

Several mechanisms contribute to the development of AMR, including acquisition of resistance genes through plasmids,^[^ ^6^ ^]^ the action of efflux pumps, decreased permeability of the bacterial membrane, enzymatic degradation of antibiotics, and structural modifications of drug targets.^[^ ^7^ ^]^ These adaptations enable bacteria to survive even high concentrations of antimicrobial drugs, rendering many current treatments ineffective. Thus, there is an urgent need to discover novel antimicrobial agents capable of overcoming these resistance mechanisms while maintaining broad‐spectrum efficacy.

**Figure 1:**
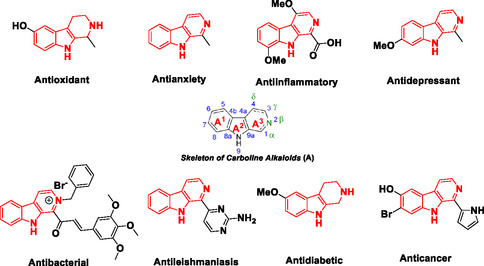
Different *β*‐carboline (shown in red) containing bioactive molecules.

Heterocyclic compounds have played a pivotal role in the pharmaceutical industry due to their broad spectrum of biological activities.^[^ ^8^ ^]^ Among these, carbolines represent a distinctive class of nitrogen‐containing heterocyclic natural products with remarkable pharmacological potential.^[^ ^9^ ^]^ Structurally, carbolines consist of a tricyclic indole moiety fuzed with a pyridine ring (designated as rings A1 and A3). Based on the saturation level of the A3 ring, carbolines are categorized into three types: unsaturated (*β*‐carbolines), semisaturated (3,4‐dihydrocarbolines), and fully saturated (1,2,3,4‐tetrahydrocarbolines). Moreover, the position of the nitrogen atom within the A3 ring further classifies them as α‐, *β*‐, γ‐, or δ‐carbolines, as illustrated in **Figure** **1**.^[^ ^10^ ^]^ To date, over 140 naturally occurring *β*‐carboline derivatives have been identified in both plant and animal sources. Notably, the fundamental *β*‐carbolines—harmala alkaloids such as harman and norharman were first isolated from Peganum harmala L. (Zygophyllaceae), a traditional medicinal plant used for centuries in North Africa and the Middle East.^[^ ^11^ ^]^

*β*‐Carbolines are pharmacologically significant due to their diverse biological properties, including antioxidant,^[^ ^12^ ^]^ antianxiety,^[^ ^13^ ^]^ antiinflammatory,^[^ ^14^ ^]^ antidepressant,^[^ ^15^ ^]^ antileishmanial,^[^ ^16^ ^]^ antidiabetic,^[^ ^17^ ^]^ anticancer,^[^ ^18^ ^]^ and antibacterial^[^ ^19^ ^]^ activities (Figure 1).

These heterocycles have also attracted considerable interest due to their ability to interact with various neurotransmitter receptors, including serotonin, dopamine, benzodiazepine, opioid, and imidazoline receptors^[^ ^20^ ^]^ as well as their capacity for DNA intercalation,^[^ ^21^ ^]^ cyclin‐dependent kinase (CDK) inhibition,^[^ ^22^ ^]^ and topoisomerase inhibition.^[^ ^23^ ^]^

**Figure 2:**
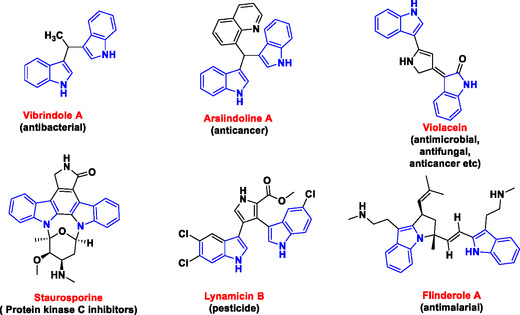
Representative examples of bisindole (highlighted in blue) bearing bioactive compounds.

Similarly, bis(Indolyl)methanes (BIMs), also known as diindolylmethanes (DIMs), have garnered considerable attention in both synthetic and pharmaceutical industries due to their presence in a wide range of biologically active compounds such as vibrindole A (antibacterial),^[^ ^24^ ^]^ Arsiindoline A(anticancer),^[^ ^25^ ^]^ Violacein (antibacterial, antifungal, and anticancer),^[^ ^26^ ^]^ Lynamicin B (pesticide),^[^ ^27^ ^]^ staurosporine (protein kinase inhibitor),^[^ ^28^ ^]^ and FlinderolesA (antimalarial)^[^ ^29^ ^]^, as depicted in **Figure** **2**.

**Figure 3:**
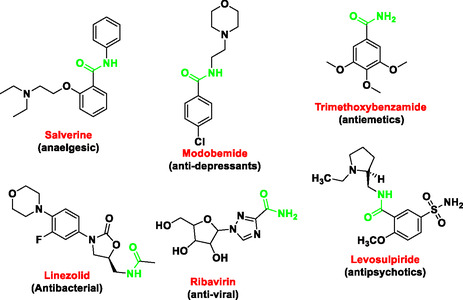
Some examples of carboxamide‐containing bioactive compounds (highlighted in green).

In addition, chemical compounds bearing carboxamide functionalities exhibit a broad spectrum of medicinal properties, including analgesic, antidepressant, antiemetic, antipsychotic, antiviral, and antibacterial activities, as illustrated in **Figure** **3**.^[^ ^30^, ^31^, ^32^, ^33^ ^–^ ^34^ ^]^ The presence of the amide linkage enhances metabolic stability and improves pharmacokinetic properties, making it a valuable structural feature in drug design and development.^[^ ^35^ ^]^ Furthermore, the α‐acylaminoamide linker imparts hydrogen bonding potential and molecular flexibility, attributes that have been successfully exploited in the development of peptidomimetic antibacterial agents.^[^ ^36^ ^]^

Given the rising concern of MDR pathogens^[^ ^37^ ^]^ such as *Staphylococcus aureus*, *E. coli*, *Pseudomonas aeruginosa*, and *Acinetobacter baumannii*, designing new hybrid molecules that combine multiple active pharmacophores into a single entity is a promising approach.^[^ ^38^ ^]^ This MH strategy can result in compounds with enhanced biological activity, multitarget potential, and the ability to overcome resistance.^[^ ^39^ ^]^ Recent advances in molecular hybridization have demonstrated the potential of combining two or more pharmacophores to create a single entity with improved potency.^[^ ^40^, ^41^, ^42^, ^43^ ^–^ ^44^ ^]^ Notably, hybrids involving clinically used antibiotics have shown promise in enhancing efficacy and overcoming resistance.^[^ ^45^, ^46^ ^–^ ^47^ ^]^ However, reports on *β*‐Carboline‐based hybrid molecules specifically designed for antibacterial activity remain underexplored.

**Figure 4:**
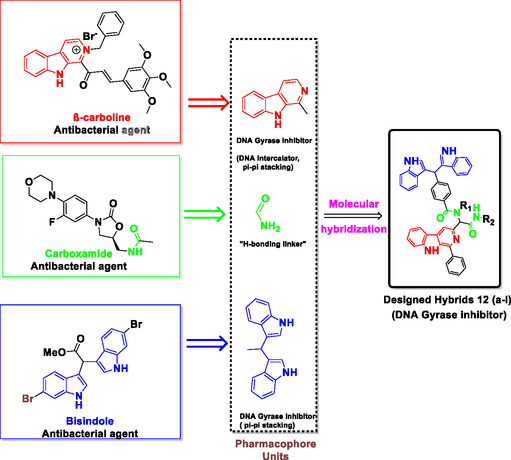
Annotated design of novel antibacterial *β*‐carboline‐{*α‐acylaminoamide*}‐bisindole hybrids.

Inspired by the biological significance of the aforementioned pharmacophores, thepresent study aimed to develop a library of novel MHs as potential antibacterial agents. These hybrids were designed (**Figure** **4**) by combining the pharmacophoric features of *β*‐carboline and bis(indolyl)methane, both of which are known to exhibit potent activity as DNA gyrase inhibitors.^[^ ^44^ ^,^ ^48^ ^]^ This rational hybridization approach was intended to enhance antibacterial efficacy against a range of Gram‐positive and Gram‐negative microorganisms. All the synthesized compounds were characterized using different spectroscopic techniques, including Fourier‐transform infrared spectroscopy, NMR, and mass spectrometry (MS). Additionally, molecular docking studies were conducted to evaluate the binding interactions of selected MHs with bacterial DNA gyrase, a well‐established antibacterial target.^[^ ^49^ ^]^ To further support these findings, density functional theory (DFT) calculations were employed to investigate the stability profiles and global reactivity parameters of the compounds.

## 2 Results and Discussion

### 2.1 Synthesis

**Scheme 1:**
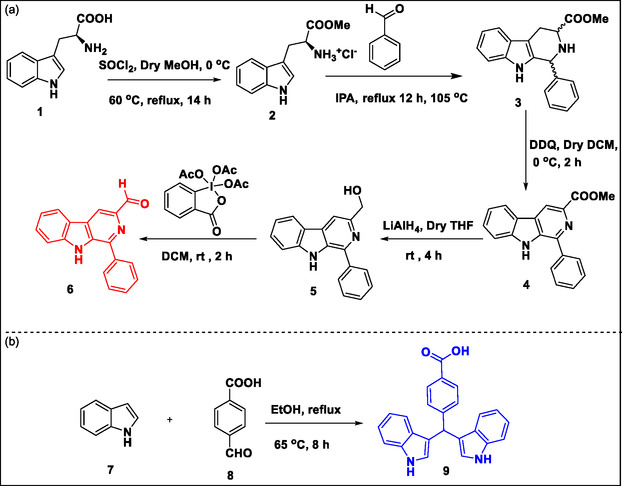
Synthetic routes for *β*‐Carboline aldehyde (**6**) and bisindole acid (**9**).

The synthetic routes adopted for the synthesis of intermediates and target compounds are illustrated in **Schemes** **1** **–**2. Initially, methyl‐1‐phenyl‐9H‐pyrido[*3,4‐b*] indole‐3‐carbaldehyde **6** and 4‐(di(1H‐indol‐3‐yl)methyl)benzoic acid **9** were synthesized using the reported method.^[^ ^50^ ^,^ ^51^ ^]^ Accordingly, for the synthesis of Precursor I (**6**) *L*‐tryptophan **1** was esterified using SOCl_2_ and dry methanol to obtain *L*‐tryptophan methyl ester **2**. The product obtained was then subjected to a Pictet Spengler Cyclization with benzaldehyde to form tetrahydro *β*‐carboline ester **3** as a diastereomeric mixture. These diastereomeric products were directly used and converted to the completely aromatized product **4**, which was further subjected to reduction with LiAlH_4_ to synthesize the respective carbinol **5**. The precursor I (**6**) was prepared by oxidation of **5** with Dess Martin Periodinane(DMP) as illustrated in Scheme 1a.

The synthesis of precursor II (**9**) involved a condensation reaction between an indole **7** and *p*‐formyl benzoic acid **8**, as shown in Scheme 1b.

### 2.2 Optimization Studies for the Target Molecular Hybrid

**Scheme 2:**
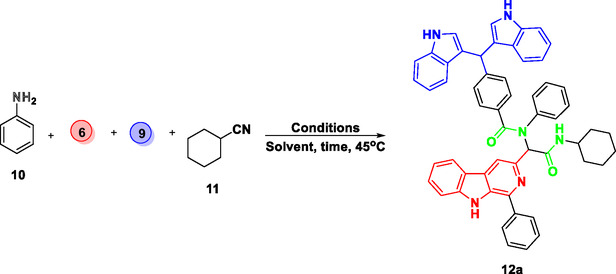
The synthetic route for the synthesis of final MHs.

Our preliminary studies employed a model reaction between Aniline **10**, **6**, **9**, and cyclohexylcyanide **11** to prepare the MH **12a** as depicted in **Scheme** **2**. The results of the optimizations that were studied for the Ugi reaction with respect to the solvent and reaction time are summarized in **Table** **1**.

**Table 1:** Results of optimization studies for **12a**.

Entry	Solvent	Time [ h]	Yield [%]a)
1	MeOH	48	73
2	EtOH	48	70
3	H_2_O	48	35
4	CH_3_CN	48	16
5	THF	48	18
6	Toulene	48	22
7	CH_2_Cl_2_	48	28
8	MeOH: CH_2_Cl_2_ (1:1)	48	68
9	MeOH: CH_2_Cl_2_ (1:2)	48	65
**10**	**MeOH: CH**_**2**_ **Cl**_**2**_ **(2:1)**	**16**	**95**

a) Isolated yield.

To optimize the Ugi four‐component reaction, a range of solvents and solvent mixtures was systematically screened. Initially, individual solvents were evaluated under identical conditions (48 h, 45 °C). The optimization study revealed that the choice of solvent significantly influences the product yield. Among the polar protic solvents tested, methanol and ethanol afforded good yields up to 73% (Table 1, entries 1 and 2). This enhanced performance may be attributed to their ability to stabilize reaction intermediates through hydrogen bonding. In contrast, water, despite being a polar protic solvent, resulted in a significantly lower yield of 35% (Table 1, entry 3). This may be attributed to the poor solubility of the organic substrates in water and the increased likelihood of side reactions such as hydrolysis.

**Scheme 3:**
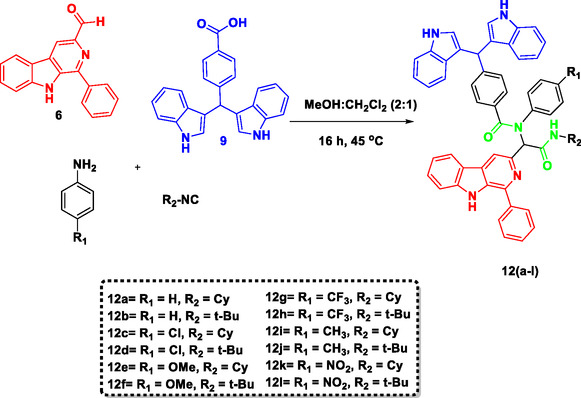
Illustrate the synthesis of β‐carboline‐{*α‐acylaminoamide*}‐ bisindole hybrids using optimized conditions.

In polar aprotic solvents such as acetonitrile (Table 1, entry 4) and THF (Table 1, entry 5), the yields were much lower (16% and 18%, respectively), attributed probably to the partial solubility of the starting materials and improper stabilization of the reaction intermediates. The reaction in toluene (Table 1, entry 6) afforded only a 22% yield, again suggesting limited compatibility due to its nonpolar nature. The yield was slightly improved by shifting to dichloromethane (Table 1, entry 7), a nonpolar aprotic solvent, although it was still far less efficient than protic solvents. Hence, a balance of polarity and good solubility in a binary solvent system was considered to be a sensible option. Accordingly, a 1:1 mixture of MeOH:CH_2_Cl_2_ offered an improved yield of 68% (Table 1, entry 8). However, increasing the proportion of dichloromethane (1:2) (Table 1, entry 9) slightly decreased the yield (65%). Interestingly, the best results were obtained in MeOH:CH_2_Cl_2_ solvent system using a 2:1 ratio (Table 1, entry 10), affording a yield of 95% with a reduced reaction time of 16 h. These results indicated that this binary solvent system not only accelerates the reaction rate but also maximizes the yield of the product. Further, these results suggest that a methanol‐rich environment not only enhances the solubility but also facilitates better stabilization of intermediates, because dichloromethane, in contrast to THF or toluene, can dissolve both polar and nonpolar reactants well and does not compete in hydrogen bonding. Hence, the optimized reaction conditions were applied to prepare a library of molecular hybrids **12a–l** (**Scheme** **3**).

### 2.3 Spectroscopic Characterization

**Figure 5:**
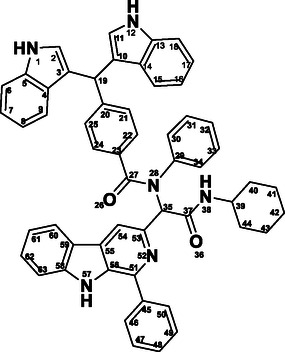
Labeled structure of the hybrid **12a**.

The structures of all synthesized molecular hybrids (12a–l) were elucidated using a combination of spectroscopic techniques, including Fourier‐transform infrared spectroscopy (FTIR), ^1^H and ^13^C NMR, and mass spectrometry (MS). As a representative example, the ^1^H NMR spectrum of hybrid 12a (**Figure** **5**) displayed characteristic signals, including a singlet at *δ* 11.45 ppm corresponding to the NH proton (H‐57) of the *β*‐carboline moiety, and a doublet at *δ* 10.87 ppm for the NH proton (H‐12). A singlet at *δ* 5.72 ppm was observed for the CH proton (H‐19) of the bisindole unit, while the cyclohexyl protons appeared as a multiplet in the range of *δ* 1.80–1.05 ppm. Furthermore, the structure of compound 12a was fully supported by its proton‐decoupled ^1^ ^3^C NMR spectrum, which exhibited characteristic signals consistent with the proposed framework. Notably, peaks at *δ* 169.89 ppm (C‐27) and *δ* 167.94 ppm (C‐37) correspond to the two amide carbonyl carbons. A distinct signal at *δ* 47.71 ppm was attributed to C‐19 of the bisindole moiety. All aromatic carbon signals appeared within the range of *δ* 145.60–111.28 ppm, while the signals between *δ* 32.06–24.32 ppm were assigned to the cyclohexyl ring carbons (C‐39 to C‐44). The IR spectrum of **12a** further showed characteristic absorption at *ν* 1631.66 cm^−1^ corresponding to the C=O functional group, whilst a strong broad absorption peak around *ν* 3270.55 cm^−1^ accounted for the NH group. Finally, the appearance of the molecular ion peak at m/z 823.9 [M + H] confirmed the assigned structure of **12a**.

### 2.4 Antimicrobial Evaluation

To assess the in vitro antibacterial activity of the synthesized hybrids, an agar‐diffusion assay was employed using the Kirby–Bauer method against Gram‐positive and Gram‐negative bacteria, and the corresponding data are provided in the supporting information. Two Gram‐positive strains, *Staphylococcus aureus, Bacillus subtilis,* and two Gram‐negative strains *Pseudomonas aeruginosa, Acinetobacter baumannii* were used to screen the antibacterial activity of the hybrids **12a–l**. The antibacterial experiments followed a completely randomized design (CRD). Each bacterial strain was tested on an individual plate, and different compounds were randomly assigned to wells in triplicate. No blocking factors (such as strain, plate, or dilution level) were used, and each treatment was considered independent. The preliminary studies (**Table** **2**), showed that the synthesized hybrids exhibit inhibition zones (ZIs) ranging between 4.3 to 22.6 mm. In the case of *Acinetobacter baumannii*, all the hybrids exhibit ZIs between 5.5 to 19.6 mm. For *Pseudomonas aeruginosa*, their ZIs range between 4.8 to 15.6 mm, while for *Staphylococcus aureus* active the ZIs range between 8.67 and 22.6 mm, respectively. Finally, in the case of *Bacillus subtilis,* all active hybrids had ZIs from 3.5 to 15.6 mm.

**Table 2:** Zone of inhibition (in mm) shown by the synthesized hybrids **12a–l**.

S.No.	Product [5 mg mL^−1^]	*Acinetobacter Baumannii*	*Pseudomonas aeruginosa* [PAO1]	*Staphylococcus Aureus* [ATCC4300]	*Bacillus Subtilis*
**12a**	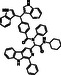	18.6 ± 0.57	12 ± 1	0	0
**12b**	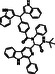	19.6 ± 0.57	15.3 ± 0.57	16.6 ± 0.57	15.6 ± 0.57
**12c**	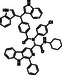	15.6 ± 0.57	15.6 ± 0.57	0	11.3 ± 0.57
**12d**	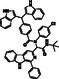	17.3 ± 0.57	13	0	0
**12e**	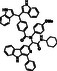	18.3 ± 0.57	12.3 ± 0.57	0	12.6 ± 0.57
**12f**	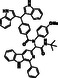	13 ± 1	0	0	0
**12g**	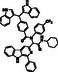	10.3 ± 0.57	16 ± 0.5	0	0
**12h**	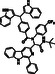	14.7 ± 0.57	10.6 ± 0.57	11.3 ± 0.57	12 ± 1
**12i**	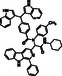	4.34 ± 0.57	6.67 ± 0.76	22.67 ± 0.57	4.34 ± 0.57
**12j**	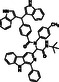	0	8.16 ± 0.76	8.67 ± 0.57	0
**12k**	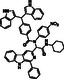	5.83 ± 0.29	4.83 ± 0.76	17.83 ± 0.76	11.34 ± 0.57
**12l**	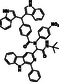	5.5 ± 0.5	10.16 ± 0.29	12.67 ± 0.57	3.5 ± 0.5
**Gentamicin** **[50 µg mL^−1^]**		0	15.6 ± 0.57	25.3 ± 0.57	28.6 ± 0.57

The minimum inhibitory concentrations (MIC) values of tested compounds **12a–l** are summarized in **Table** **3**. Five compounds, **12g**, **12h**, **12e**, **12d**, and **12b** showed the highest activity against *Acinetobacter baumannii* and *Pseudomonas aeruginosa* with MIC values ranging between (302–308 µg mL^−1^). Whereas the three compounds (**12a**, **12c**, and **12f**) showed relatively low activity (MIC = 617–621 µg mL^−1^) against the same two strains, while the four compounds (**12i**, **12j**, **12k,** and **12l**) displayed very low activity (MIC ≥ 1172 µg mL^−1^).

**Table 3:** Antibacterial evaluation of the synthesized hybrids **12a–l**.

S.No.	Product	*Acinetobacter* *Baumannii*	*Pseudomonas* *aeruginosa* [PAO1]	*Staphylococcus* *Aureus* [ATCC4300]	*Bacillus* *Subtilis*
**12a**	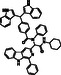	625	625	2500	1250
**12b**	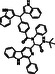	303	303	1242	303
**12c**	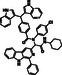	617	617	2495	1243
**12d**	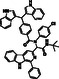	307	623	2498	1245
**12e**	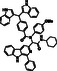	306	306	2497	1244
**12f**	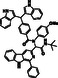	619	1239	2479	1239
**12g**	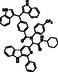	303	303	2493	1246
**12h**	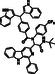	302	302	2498	1244
**12i**	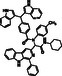	1170	618	2492	1246
**12j**	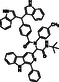	1248	624	2496	1248
**12k**	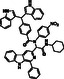	1250	624	2500	1250
**12l**	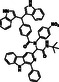	1245	622	2490	1245
**6**	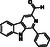	623	623	2476	1224
**9**	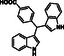	307	623	146	146
**Gentamicin**		–	3.34–55.87	3.82–52.05	3.82–52.05

a) DMSO was used as the control solvent, and MIC values are represented in  μg mL^−1^.

In the case of *Acinetobacter baumannii*, three compounds (12b, 12g, and 12h) exhibited nearly identical and the highest potency among all synthesized hybrids, with MIC values ranging from 302 to 303 µg mL^−1^. These activities surpass those of their precursors, compound I (6) (MIC = 623 µg mL^−1^) and compound II (9) (MIC = 308 µg mL^−1^), with 12h showing the best activity. This result highlights the significance of molecular hybridization in enhancing antibacterial efficacy. Compounds 12d and 12e showed MICs of 306–307 µg mL^−1^, roughly doubling the activity compared to precursor I (6). Compounds 12i, 12k, and 12l exhibited low activity. Against *Pseudomonas aeruginosa* 12b, 12e, 12g, and 12h demonstrated improved activity (MIC = 302–306 µg mL^−1^) over precursors I and II (623 µg mL^−1^), with 12h being the most active. The remaining compounds showed MICs similar to the precursors (617–625 µg mL^−1^).

Against the Gram‐positive bacterium *Staphylococcus aureus*, compound 12b showed the highest, though weak, activity among the synthesized hybrids (MIC = 1242 µg mL^−1^), outperforming precursor I (6) (MIC = 2476 µg mL^−1^) but exhibiting significantly lower activity than precursor II (MIC = 147 µg mL^−1^). All other compounds of the series exhibited very low or no activity for the same bacteria (MIC ≥ 1242 µg mL^−1^). For *Bacillus subtilis*, compound 12b exhibited the highest activity among all compounds (MIC = 303  µg mL^−1^), outperforming precursor I (MIC = 2476 µg mL^−1^) but less active than precursor II (MIC = 146 µg mL^−1^). The remaining compounds showed similarly weak activity with MICs ≥ 1245 µg mL^−1^.

Compounds 12g, 12h, and 12b showed the most consistent activity across the Gram‐negative strains (Table 3). These results support the design rationale of combining *β*‐carboline, bisindole, and *α*‐acylaminoamide pharmacophores to enhance antibacterial efficacy. The observed consistency with the hybrid scaffold hypothesis, particularly against Gram‐negative bacteria, highlights the advantage of integrating multiple interaction modes within a single molecule. Although no clear trend was observed across all analogs, some differences in activity may be attributed to structural variations.

### 2.5 Structure–Activity Relationship (SAR)

The structure–activity relationship (SAR) analysis of the synthesized *β*‐carboline–*α*‐acylaminoamide–bisindole hybrids **12a–l** revealed that compounds **12b**, **12g**, and **12h** demonstrated relatively higher antibacterial activity, particularly against Gram‐negative strains such as *Acinetobacter baumannii* and *Pseudomonas aeruginosa*. Compounds **12g** and **12h** contain strongly electron‐withdrawing trifluoromethyl (–CF_3_) groups on the phenyl ring, which probably enhance bacterial membrane permeability and target binding via increased lipophilicity. Notably, compound **12b**, which possesses an unsubstituted phenyl ring, also exhibited comparable potency, highlighting the significant intrinsic contribution of the hybridized scaffold to biological activity. This indicates that the hybrid framework itself plays a dominant role in driving antibacterial activity, even in the absence of electron‐modifying groups. Furthermore, hybrids bearing cyclohexyl substituents generally outperformed those with tert‐butyl groups, possibly due to improved accommodation within hydrophobic enzyme pockets. In the case of *Acinetobacter baumannii*, the compounds containing a *p*‐trifluoromethyl, *p*‐chloro, *p*‐methoxy substituents and unsubstitution on the phenyl ring showed good antibacterial activity. For *Pseudomonas aeruginosa* (PAO1), compounds containing an unsubstituted phenyl ring with a tert‐butyl group showed comparable activity to those with *p*‐trifluoromethyl and *p*‐methoxy substituents. The compound having an unsubstituted phenyl ring with a tert‐butyl group was most active against *Staphylococcus aureus*. In the case of *Bacillus subtilis*, the compound bearing an unsubstituted phenyl ring with a tert‐butyl group exhibited the highest activity among all the derivatives, whereas all other compounds having *p*‐chloro, *p*‐methoxy, *p*‐trifluoromethyl, *p*‐methyl, and *p*‐nitro groups on the phenyl ring showed comparable and significantly lower activity.

### 2.6 Docking Studies

DNA gyrase acts as a key therapeutic target in developing antibacterial drugs.^[^ ^52^ ^]^ Its presence in bacteria and absence in humans allows for its selective inhibition without harming the host cells.^[^ ^49^ ^]^ Numerous research teams have published information on synthesizing, characterizing, and biological assessment of peptides containing indole as DNA gyrase inhibitors.^[^ ^53^ ^]^ Moreover, the antibacterial activities of *β*‐carbolines are well‐established in the literature.^[^ ^54^ ^]^ Therefore, Molecular docking was conducted using the Schrödinger suite (2022–1 release) with calculations based on the OPLS4 forcefield. First, the crystal structure of DNA gyrase (PDB ID: 6KZV) was downloaded from the Protein Data Bank (PDB) and was prepared with protein purification methods. Before moving on to the docking of our compounds, the docking protocol was validated by redocking the crystallized ligand. The computed root mean square deviation (RMSD) value of 1.4 Å between the crystallographic (Green colored) and docked pose (orange‐colored) of the original ligand indicated good reproducibility, verifying the docking protocol (Figure S56, Supporting Information).

### 2.7 Docking Results

**Figure 6:**
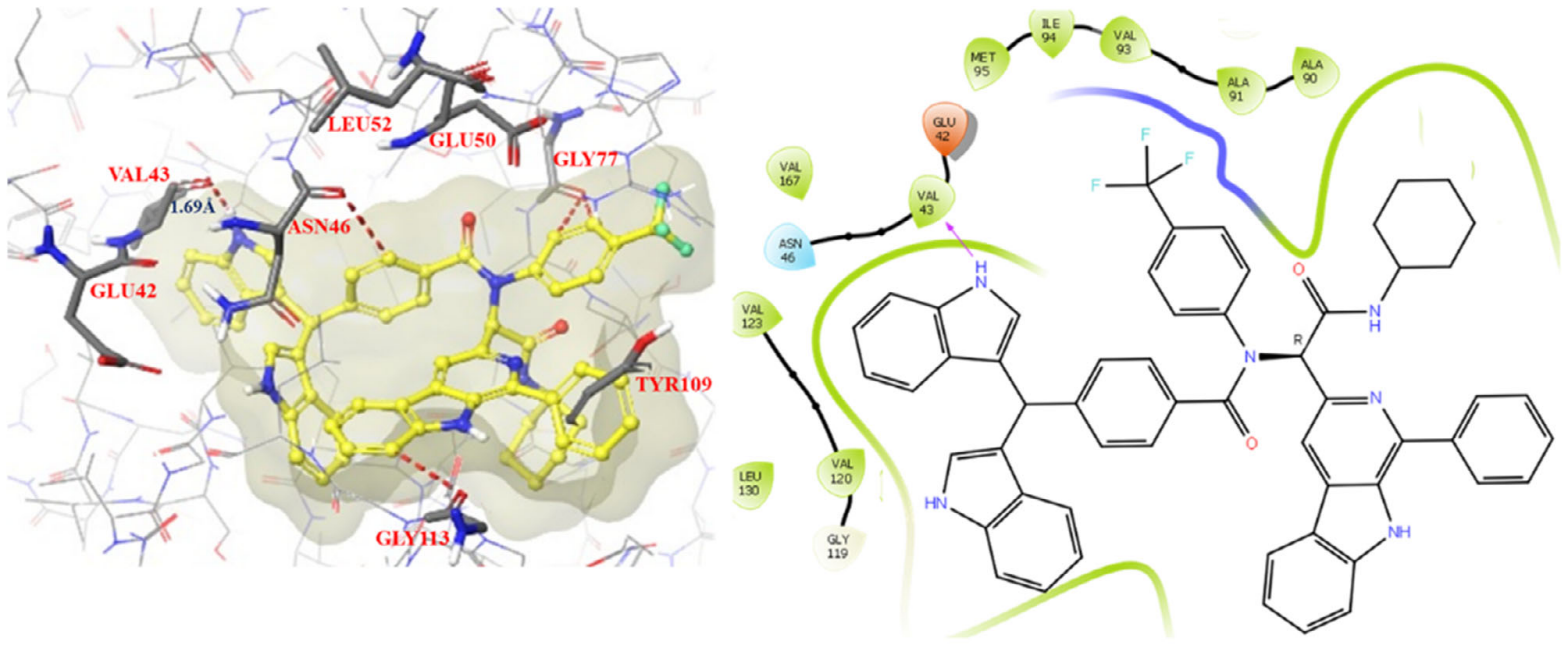
Docking interactions of compound **12g** in the active site of DNA gyrase (PDB ID: 6KZV).

**Figure 7:**
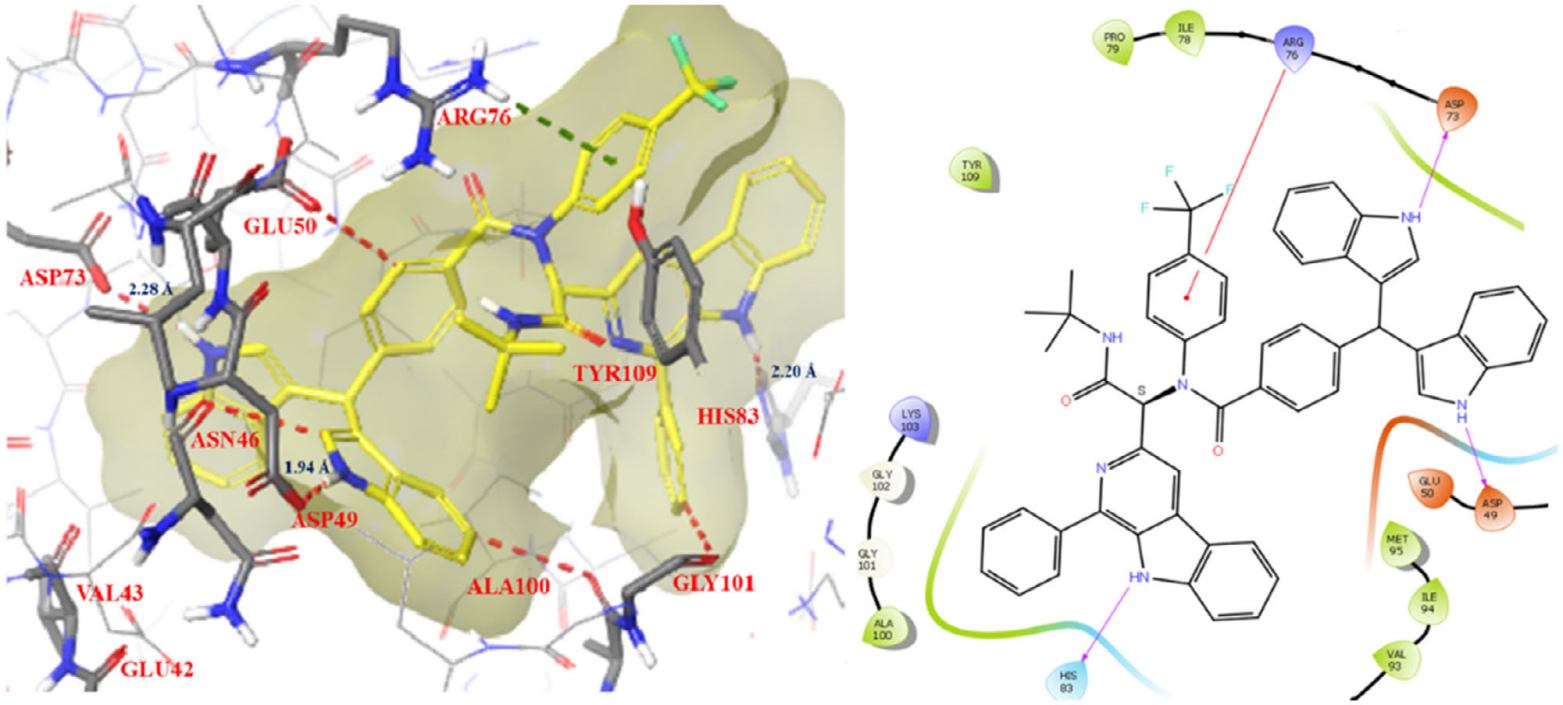
Docking interactions of compounds **12h** in the active site of DNA gyrase (PDB ID: 6KZV).

Two representative compounds **(12g** and **12h)** were docked in the active site of the DNA gyrase (PDB ID: 6KZV) to explore their binding orientations, and their docked complexes are shown in **Figure** **6** and **7**. The best pose for each protein–ligand complex was chosen based on docking scores, glide scores, and XP energy (**Table** **4**). A closer inspection of these Figure revealed that both **12g** and **12h** interact effectively in the enzyme's active site, stabilized by hydrophobic and hydrophilic amino acid residues.

**Table 4:** Docking score and key interactions of the most active compounds (**12g** and **12h**).

Compound	Docking Score [ Kcal mol^−1^]	XPScore	H‐bonding interactions	Aromatic‐Hbondinginteractions	Hydrophobicinteractions
**12g**	−5.20	−5.55	Val 43	Asn 46, Gly 113,and Gly 77	
**12h**	−5.55	−5.89	Asp 73,Asp 49,and His 83	Glu 50, Ala100,Gly 101, andAsn 46	Arg 76

Compound **12h** formed H‐bonding interactions with Asp 73, Asp 49, and His 83 within bonding distances of 2.28, 1.94, and 1.73 Å, respectively, showing tight binding of the compound within the enzyme active site. A hydrophobic *π*‐cation interaction between the aromatic ring and the amino group of Arg76 is also observed. Since a large part of the compound **12h** consists of aromatic rings, various aromatic‐hydrogen bonding interactions of this part and surrounding residues are observed, such as with Glu50, Ala100, Gly 101, and Asn 46 (Table 4). Similarly, compound **12g** showed H–bonding interaction with Val 43 (bonding distance of 1.43 Å) and aromatic‐hydrogen bonding interactions with Asn 46, Gly 113, and Gly 77 amino acid residues.

### 2.8 DFT Analysis

DFT was utilized to investigate Frontier molecular orbitals (FMOs) and the global reactivity of the synthesized compounds.

### 2.9 Structural Optimization Energy

**Figure 8:**
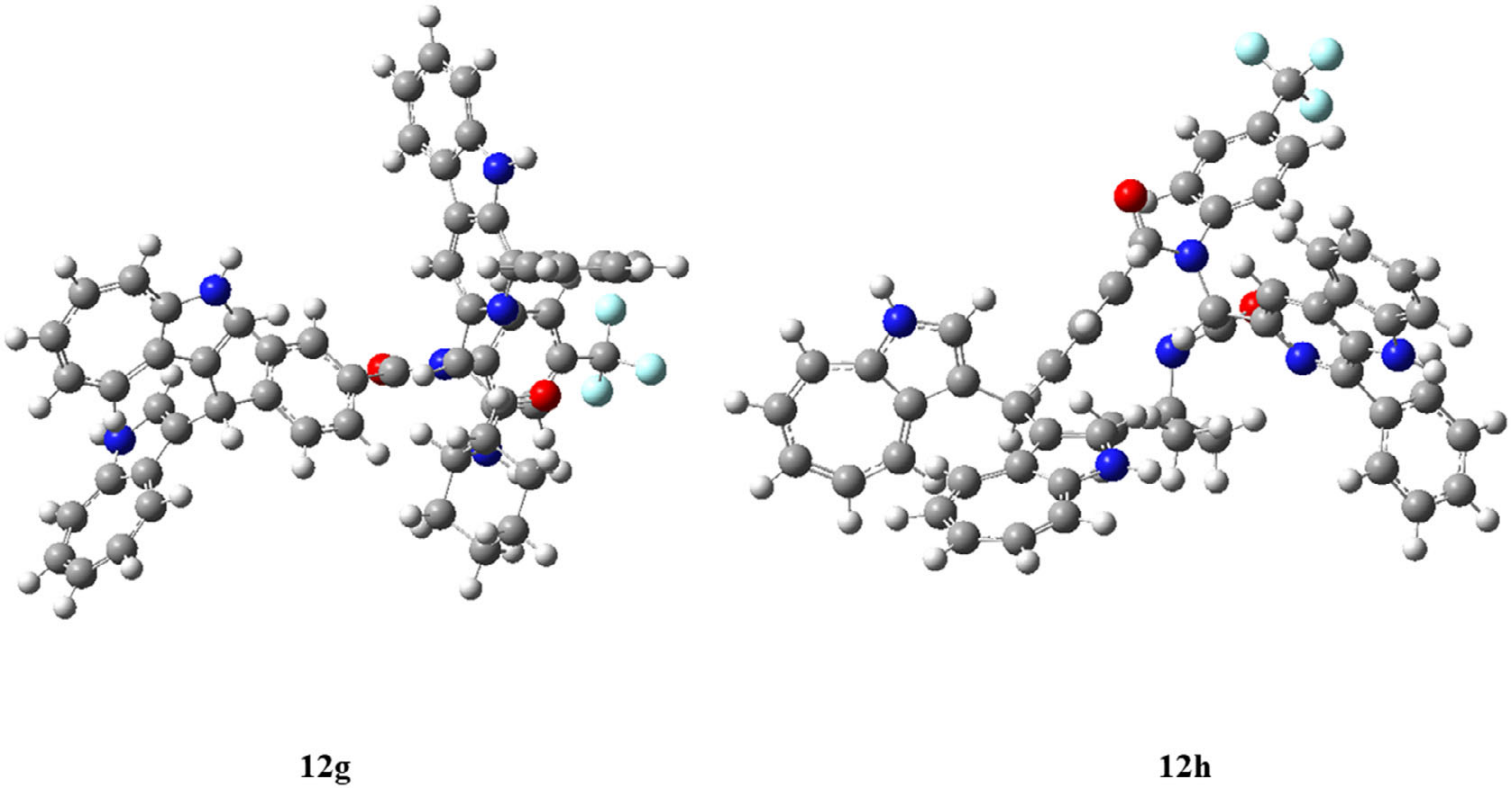
Illustrate the optimized structure of the compound **12g** and **12h** using DFT/B3LYP/6‐31++G (d,p).

The DFT studies were carried out on selected potent compounds (**12g** and **12h**) among the series for their structure optimization energy. The B3LYP/6‐31++G (d, p) method was applied for DFT investigation^[^ ^55^ ^]^. The optimized structures of compounds **12g** and **12h** are illustrated in **Figure** **8**.

The dipole moment and electronic energy of the compounds were calculated under vacuum media, and the results are summarized in **Table** **5**. The compounds **12g** and **12h** exhibited a dipole moment of 9.06 and 8.38 Debye, respectively. The higher dipole moment of **12g** suggested it was more polar than **12h**. Furthermore, the optimized energies of **12g** and **12h** were found to be −3017.08 and −2939.64 Hartree, respectively.

**Table 5:** The dipole moment and the electronic energy using DFT/B3LYP/6‐31++G (d, p).

compound	Dipole moment [vacuum] [Debye]	Electronic energy [vacuum] [Hartree]
**12g**	9.06	−3017.08
**12h**	8.38	−2939.64

### 2.10 Frontier Molecular Orbital (FMO) Analysis

**Figure 9:**
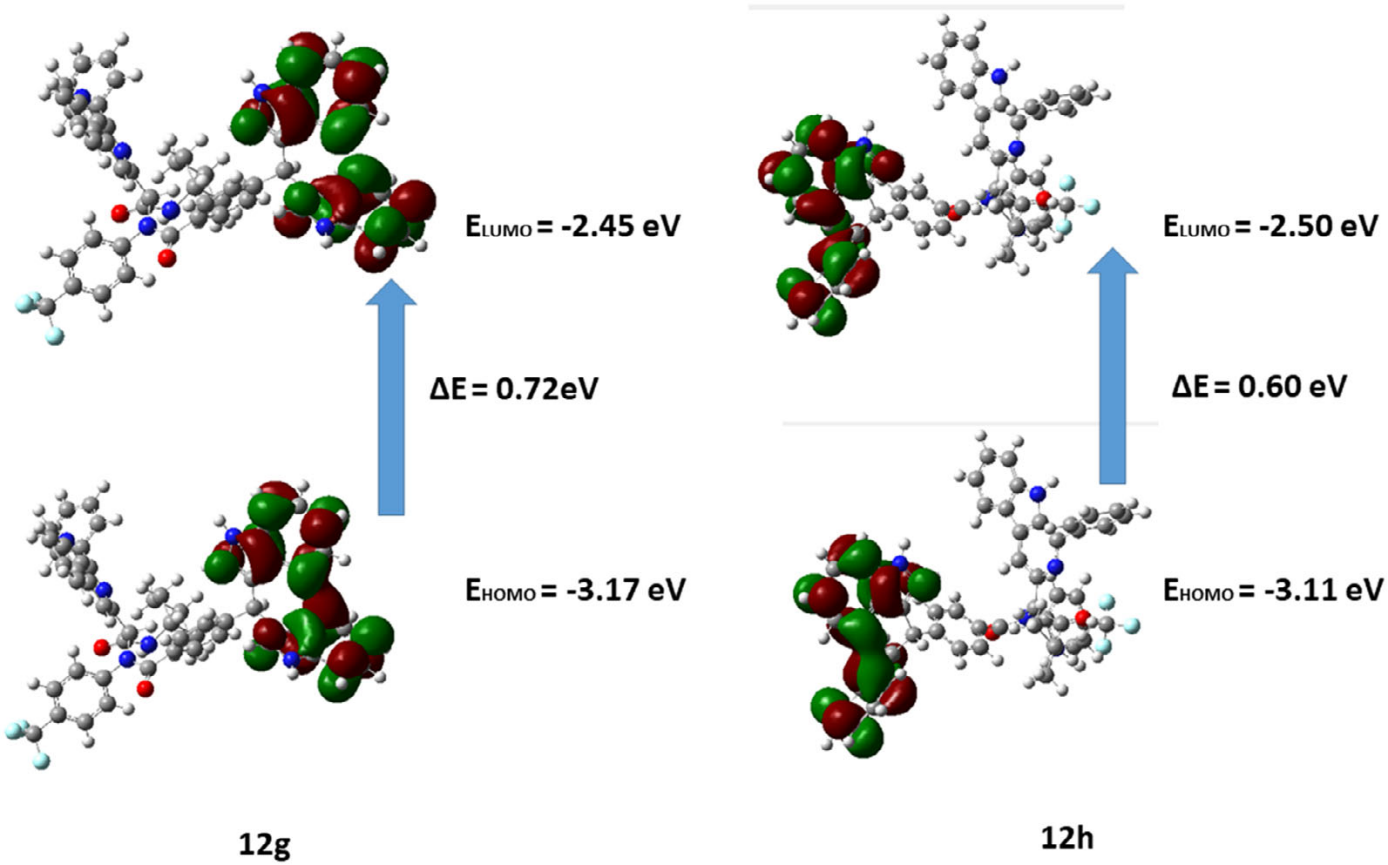
Illustration of the FMOs of compounds **12g** and **12h**.

As a fundamental tool for determining a molecule's electronic properties and chemical stability, FMOs, such as the highest occupied molecular orbital (HOMO) and the lowest unoccupied molecular orbital (LUMO), play a pivotal role.^[^ ^56^, ^57^ ^–^ ^58^ ^]^ An HOMO can be defined as a loosely bound electron orbital at the highest energy, while a LUMO can be defined as an unoccupied electron orbital at the lowest energy.^[^ ^59^ ^]^ It is essential to identify the energies of these orbitals to assess the structural and reactivity properties of a compound. Accordingly, the FMO calculations of all compounds were performed at the DFT level, and the results are depicted in **Table** **6**. Compound **12h** (E_HOMO_ = −3.11 eV) was predicted to have the higher energy of its HOMO as well as its LUMO (E_LUMO_ = −2.50 eV). An energy gap is defined as the difference between the HOMO and LUMO energies, and a smaller gap indicates greater reactivity, whereas a larger gap indicates greater stability.^[^ ^60^, ^61^ ^–^ ^62^ ^]^ Compound **12h**, with the lower energy gap (Δ*E* = 0.60 eV), was predicted to be more reactive. Also, the charge density of the HOMO and LUMO in these compounds was distributed across the entire molecule, as depicted in **Figure** **9**.

**Table 6:** The FMO energies of **12g** and **12h**.

Propertiescode	E_HOMO_ [ eV]	E_LUMO_ [eV]	ΔE [ eV]
**12g**	−3.17	−2.45	0.72
**12h**	−3.11	−2.50	0.60

### 2.11 Calculation of Global Reactivity Properties

The result presented in **Table** **7** demonstrates the global reactivity parameters such as ionization potential (*I*), electron affinity (*A*), hardness (*η*), electronegativity (*χ*), softness (*S*), chemical potential (μ), and electrophilicity index (*ω*) of most active compounds **12g** and **12h**.^[^ ^63^, ^64^ ^–^ ^65^ ^]^

**Table 7:** The calculated global reactivity properties of the **12g** and **12h**.

PropertiesCode	I [ eV]	A [ eV]	*χ* [ eV]	*η* [ eV]	S [ eV^−1^]	µ [ eV]	ω [ eV]
**12g**	3.17	0.72	1.63	0.36	0.32	−1.63	3.69
**12h**	3.11	0.60	1.60	0.30	0.33	−1.60	4.22

Molecules are characterized by their ionization potential (*I*) and electron affinity (*A*), which reflect the ability to lose and gain electrons, respectively.^[^ ^66^ ^]^ The lower the ionization potential, the easier it is to remove electrons, whereas a higher electron affinity indicates a greater tendency to accept electrons.^[^ ^67^ ^]^ Accordingly, compound **12h** with the lower I value (I = 3.11 eV) was predicted to be the stronger base, while compound **12g** with the higher A value (A = 0.72 eV) was predicted to be the stronger electrophile. Electronegativity (*χ*) displays the potential of a molecule to attract electrons toward itself.^[^ ^68^ ^]^ Compound **12g** (1.63 eV) displayed the highest electronegativity when compared to **12h**. Chemical potential (μ) is linked to electronegativity (*χ*) and measures the electrons’ propensity to be attracted toward each other within a molecule.^[^ ^69^ ^]^ Compound **12g** (−1.63 eV) displayed a lower chemical potential relative to **12h**.

Global hardness (*η*) measures a molecule's resistance to charge transfer, while global softness (*S*) indicates its tendency to facilitate charge transfer.^[^ ^70^ ^]^ It is preferable for molecules to have a lower global hardness and a higher global softness, as these properties enhance the ability of the molecule to transfer charge to adjacent biomolecules, thus improving molecular interactions. The compound **12h** (*η *= 0.30 eV and S = 0.33 eV^−1^) was computationally found to show better interaction with nearby molecular or biomolecules as compared to **12g**.

The Electrophilicity Index refers to the ability to attract electrons, which is reflected in the energy required to stabilize the molecule when it interacts with electrons in the environment that are nearby.^[^ ^71^ ^]^ The calculated results demonstrated that compound **12h** (4.22 eV) has a higher electrophilicity Index.

## 3 Conclusion

In summary, a new series of *β*‐carboline‐{*α*‐acylaminoamide}‐bisindole hybrids **12a–l** has been synthesized via a one‐pot Ugi reaction and subjected to antibacterial activity against Gram‐positive and Gram‐negative strains, followed by computational investigations. Among these compounds, compounds **12g**, **12h**, and **12b** exhibited notable activity, particularly against MDR Gram‐negative strains. Although the observed activity was moderate, the consistent enhancement over precursor compounds indicates the promise of molecular hybridization in improving antibacterial efficacy, especially against Gram‐negative strains. The biological performance of **12g**, **12h,** and **12b** was further supported by molecular docking and DFT studies, indicating favorable binding interactions and electronic characteristics. In addition to their biological efficacy, the synthetic strategy employed is highly cost‐effective and operationally simple, utilizing a one‐pot multicomponent protocol under mild conditions. This approach minimizes purification steps, reduces synthetic effort, and shortens the overall process compared to conventional stepwise synthesis. It also reduces waste and reaction time, and facilitates reproducibility and scalability, making it an attractive route for the rapid generation of diverse molecular scaffolds. While the activity was moderate, the hybrid scaffold presents a novel framework that may serve as a basis for future optimization for antibiotic development. This study primarily focused on the synthesis and antibacterial screening of hybrids against Gram‐positive and Gram‐negative bacteria. Future work may include toxicological assessments and environmental safety studies to further explore the potential of these compounds.

## 4 Experimental Section

4.1

4.1.1

##### Chemistry

Every chemical has been bought from TCI and Sigma Aldrich. The solvents are bought from commercial sellers and subjected to a simple distillation process before use. Thin‐layer chromatography (TLC) using Fluorescence F254 and UV imaging on silica gel plates was employed for monitoring the synthesis and purity of the built compounds.For flash chromatography, silica gel with a mesh size ranging from 230 to 400 was used. Using a Bruker AV‐500 spectrophotometer, ^1^H NMR and ^13^C NMR spectra were obtained at 500 and 126 MHz, respectively. Coupling constants *J* values are expressed in Hz or hertz. In terms of an internal standard (tetramethylsilane), chemical shifts (*δ*) were expressed in parts per million. Singlet (s), doublet (d), triplet (t), quartet (q), and multiple (m) were the terms used to characterize proton coupling patterns. Each first‐order splitting pattern was assigned according to how the multiplet showed up. Multiplet (m) denotes splitting patterns that were difficult to interpret. The Infrared spectral data of the compounds were obtained using the Perkin–Elmer Spectrum Version FTIR instrument. Using a Waters MicromassQ‐ToFMicro spectrometer, spectra were obtained. Melting points were determined by using the sealed capillary tube on an SMP30 melting point apparatus.

##### General Procedure for the Preparation of Precursor I (6)

To a suspension of L‐tryptophan **1** (500 mg, 2.44 mmol) in dry methanol (40 mL), thionyl chloride (4.4 mL, 4.89 mmol) was added dropwise at 0 °C and the reaction mixture was stirred for 16 h at 65 °C.^[^ ^72^ ^]^ After the consumption of the starting material (monitored by TLC), the excess solvent was removed under a rotary evaporator, and the crude product was codistilled with methanol (2 × 15 mL) to obtain a white solid hydrochloride salt of tryptophan ester **2** with an excellent yield of 98%. To a suspension of compound 2 (500 mg, 1.96 mmol) in isopropyl alcohol (30 mL),benzaldehyde (3 mL, 2.36 mmol) was added under an inert atmosphere, and the reaction mixture was stirred for 16 h at 110 °C. After completion of the reaction (monitored by TLC), the solvent was evaporated under a rotary evaporator, basified with a saturated solution of potassium carbonate (20 mL), and a crude mixture was extracted with ethyl acetate (3 x 20 mL). The organic extract was washed with brine (20 mL), dried over anhydrous Na_2_SO_4_, and concentrated under vacuum. The resulting diastereomeric mixture **3** was directly used for the next step without any further purification.

##### Synthesis of Methyl‐1‐Phenyl‐9H‐Pyrido[3,4‐b]indole‐3‐ Carboxylate (4)

To a solution of **8** (500 mg, 1.58 mmol) in dry CHCl_3_ (25 mL) under inert conditions 2,3‐dichloro‐5,6‐dicyano‐1,4‐benzoquinone (DDQ) (718 mg, 3.16 mmol) was added portion‐wise for 5 min at 0 °C, and the reaction mixture was continued to stir for 2 h at 0 °C.^[^ ^73^ ^]^ After the completion ofthe reaction (monitored by TLC), the reaction mixture was quenched with a saturated solution of NaHCO_3_, and the crude product was extracted with ethylacetate (3 × 20 mL). The organic extract was washed with brine (20 mL), dried over anhydrous Na_2_SO_4_,and concentrated under vacuum. The residue was purified by flash chromatography (silica gel 230–400 mesh) using ethyl acetate/hexane as the eluents to obtain intermediate **4** as a white solid with a good yield of 85%.

##### Synthesis of Methyl‐1‐Phenyl‐9H‐Pyrido[3,4‐b]indole‐3‐Carbaldehyde (6)

To a solution of compound **4** (300 mg, 1 mmol) in dry THF (25 mL) under inert conditions, LiAlH_4_ (151 mg, 4 mmol) was added portion‐wise at −5 to 0 °C for 30 min, and the reaction mixture was turned into a light yellow, clear solution. Further, the reaction mixture was continued stirring for 4 h at 25 °C after completion of the reaction (monitored by TLC), a new fluorescent spot was observed, and the excess of LiAlH_4_ was quenched by adding a saturated solution of Na_2_SO_4_ · 10H_2_O until the bubbling stopped. The reaction mixture was filtered through Celite, washed with excess THF, and concentrated under vacuum. The resulting crude product **5** was directly used for the next step without any further purification.^[^ ^69^ ^]^ To a solution of crude product **5** (200 mg, 0.72 mmol) in dry CHCl_3_ (20 mL), Dess‐Martin periodinane (an oxidizing agent) (467 mg, 1.1 mmol) was added, and the reaction mixture was continued to stir for 2 h at 25 °C. After completion of the reaction (monitored by TLC), the reaction mixture was washed with water (2 × 50 mL), and the CHCl_3_ layer was extracted and dried over anhydrous Na_2_SO_4_ and concentrated under vacuum. The resulting crude product was purified by flash chromatography (silica gel 230–400 mesh) using ethyl acetate/hexane to afford compound **6** with high purity and witha yield of 78%. The structure of the product was characterized by using NMR (^1^H and ^13^C) spectroscopy and MS.

##### Synthesis of 4‐(di(1H‐Indol‐3‐Yl)methyl) Benzoic Acid (9)

To a solution of 4‐formyl benzoic acid **8** (500 mg, 3.32 mmol) in absolute ethanol (15 mL), indole **7** (978 mg, 8.30 mmol) was added, and the reaction mixture was stirred for 8 h at 65 °C. After the completion of the reaction (monitored by TLC), excess of the solvent was removed using a rotary evaporator, and unreacted traces of 4‐formyl benzoic acid were removed with washings by the saturated solution of NaHCO_3_. Crude product was extracted with ethylacetate (3 × 20 mL). The organic extract was washed with brine (20 mL), dried over anhydrous Na_2_SO_4_, and concentrated under vacuum. The resulting crude product was purified by flash chromatography (silica gel 230–400 mesh) using ethyl acetate/hexane to afford compound **9** with high purity and with an overall good yield of 85%. The structure of the product was characterized by using NMR (^1^H and ^13^C) spectroscopy, IR spectroscopy, and MS.

##### General Procedure for the Synthesis of Ugi Products (*β*‐Carboline‐{*α‐Acylaminoamide*}‐Bis‐Indole Hybrids) 12a–l

Methyl‐1‐phenyl‐9H‐pyrido[*3,4‐b*]indole‐3‐carbaldehyde **1** (1 mmol) was dissolved in a minimum volume of dry MeOH:CH_2_Cl_2_ (2:1), then the respective substituted amine **3** (1 mmol) was added, and the reaction mixture was stirred for 15 min at 45 °C. Thereafter, 4‐(di(1H‐indol‐3‐yl)methyl)benzoic acid **2** (1 mmol) and respective isocyanide **4** (1 mmol) were added sequentially to the flask, and the reaction mixture was stirred at 45 °C for 24 h (Scheme 1). The progress of the reaction was confirmed by TLC in 50% EtOAc/n‐Hexane as the eluent. After completion of the reaction, the crude was washed with water (3 × 10 mL) and extracted with EtOAc. The organic layer was separated, dried over anhydrous Na_2_SO_4_, and concentrated under a rotary evaporator. The crude product was purified by column chromatography (silica gel 100–200 mesh) using EtOAc/n‐hexane as the eluents to afford a new library of respective *β*‐carboline‐{*α‐acylaminoamide*}‐bisindole in good yields.

##### N‐(2‐(cyclohexylamino)‐2‐Oxo‐1‐(1‐Phenyl‐9H‐Pyrido[3,4‐b]indol‐3‐Yl)ethyl)‐4‐(di(1H‐Indol‐3‐Yl)methyl)‐N‐Phenylbenzamide (12a)

Light pink solid, Yield (95%), m.p 189–190 °C. ^**1**^ **H NMR** (500 MHz, DMSO‐d6) *δ* 11.45 (s, 1H), 10.80 (d, *J* = 1.5 Hz, 2H), 8.34 (d, *J* = 7.8 Hz, 1H), 8.18 (d, *J* = 7.9 Hz, 1H), 7.96 (s, 1H), 7.93 (d, *J* = 7.2 Hz, 2H), 7.62–7.57 (m, 3H), 7.54–7.49 (m, 2H), 7.32 (d, *J* = 8.1 Hz, 2H), 7.24–7.17 (m, 5H), 7.15–7.11 (m, 4H), 7.02 (t, *J* = 7.5 Hz, 2H), 6.94–6.87 (m, 3H), 6.83 (t, *J* = 7.5 Hz, 2H), 6.62 (s, 3H), 5.72 (s, 1H), 3.74 (dd, *J* = 10.9, 7.1 Hz, 1H), 1.80 (d, *J* = 9.5 Hz, 1H), 1.69 (d, *J* = 10.0 Hz, 1H), 1.60 (dd, *J* = 19.1, 9.0 Hz, 2H), 1.50 (dd, *J* = 8.8, 3.9 Hz, 1H), 1.25 (d, *J* = 12.0 Hz, 2H), and 1.18–1.05 (m, 3H). ^**13**^ **C NMR** (126 MHz, DMSO‐d6) *δ* 169.89, 167.92, 145.60, 143.92, 141.25, 141.16, 140.61, 137.89, 136.41, 134.39, 131.70, 130.16, 129.55, 128.54, 128.42, 128.18, 127.80, 127.34, 127.31, 126.36, 126.13, 123.44, 121.35, 120.74, 120.60, 119.52, 118.92, 118.05, 117.44, 114.25, 112.31, 111.28, 66.12, 47.71, 32.06, 31.94, 25.09, 24.31, and 24.27. **MS (ESI) of [C**_**55**_ **H**_**46**_ **N**_**6**_ **O**_**2**_ **+H]** ^**+**^ **(m/z)**: 823.9; Calcd: 822.4. **IR ( cm** ^**−1**^)3270.55, 2926.67, 1631.660, 1450.86, and 743.54.

##### N‐(2‐(tert‐Butylamino)‐2‐Oxo‐1‐(1‐Phenyl‐9H‐Pyrido[3,4‐b]indol‐3‐Yl)ethyl)‐4‐(di(1H‐Indol‐3‐Yl)methyl)‐N‐Phenylbenzamide (12b)

Light orange solid, Yield (84%), m.p 178–180 °C. ^**1**^ **H NMR** (500 MHz, DMSO‐d6) *δ* 11.45 (s, 1H), 10.80 (d, *J* = 1.5 Hz, 2H), 8.19–8.16 (m, 2H), 7.98 (s, 1H), 7.95 (d, *J* = 7.3 Hz, 2H), 7.62–7.57 (m, 3H), 7.52 (dt, *J* = 13.6, 7.4 Hz, 2H), 7.32 (d, *J* = 8.1 Hz, 2H), 7.25—7.19 (m, 5H), 7.14 (dd, *J* = 7.8, 4.9 Hz, 4H), 7.02 (t, *J* = 7.5 Hz, 2H), 6.94 (t, *J* = 7.5 Hz, 2H), 6.88 (t, *J* = 7.3 Hz, 1H), 6.83 (t, *J* = 7.5 Hz, 2H), 6.63 (s, 2H), 6.59 (s, 1H), 5.73 (s, 1H), and 1.25 (s, 9H). ^**13**^ **C NMR** (126 MHz, DMSO‐d6) *δ* 169.77, 168.11, 145.61, 144.30, 141.30, 140.49, 137.89, 136.41, 134.42, 131.66, 130.65, 130.07, 129.59, 128.56, 128.43, 128.16, 127.87, 127.34, 126.37, 126.09, 123.45, 121.29, 120.89, 120.75, 120.58, 119.52, 119.02, 118.93, 118.06, 117.45, 117.11, 114.02, 112.34, 111.29, 66.43, 50.22, and 28.32. **MS (ESI) of [C**_**53**_ **H**_**44**_ **N**_**6**_ **O**_**2**_ **+H]** ^**+**^ **(m/z)**: 797.9; Calcd: 796.4. **IR ( cm** ^**−1**^) 3430.90, 2250.73, 2125.03, 1658.68, 1377.94, 1233.33, 1025.75, 824.44, 763.41, and 624.59.

##### N‐(4‐Chlorophenyl)‐N‐(2‐(cyclohexylamino)‐2‐Oxo‐1‐(1‐Phenyl‐9H‐Pyrido[3,4‐b]indol‐3‐Yl)ethyl)‐4‐(di(1H‐Indol‐3‐Yl)methyl)benzamide (12c)

Pink solid, Yield (93%), m.p 174–176 °C. ^**1**^ **H NMR** ( 500 MHz, DMSO‐d6) *δ* 11.52 (s, 1H), 10.82 (s, 2H), 8.36 (d, *J* = 7.7 Hz, 1H), 8.24 (d, *J* = 7.9 Hz, 1H), 8.02 (s, 1H), 7.94 (d, *J* = 7.2 Hz, 2H), 7.64 (dd, *J* = 16.5, 8.1 Hz, 3H), 7.61–7.52(m, 2H), 7.37 (d, *J* = 8.1 Hz, 2H), 7.32–7.26 (m, 2H), 7.26–7.19 (m, 4H), 7.16 (d, *J* = 7.9 Hz, 2H), 7.07 (t, *J* = 7.5 Hz, 2H), 7.01 (d, *J* = 8.8 Hz, 2H), 6.89 (t, *J* = 7.5 Hz, 2H), 6.74 (s, 2H), 6.69 (s, 1H), 5.79 (s, 1H), 3.80–3.73 (m, 1H), 1.84 (d, *J* = 9.2 Hz, 1H), 1.72 (d, *J* = 11.9 Hz, 1H), 1.70–1.61 (m, 2H), 1.55 (d, *J* = 13 Hz, 1H), and 1.32–1.05(m, 5H). ^**13**^ **C NMR** (126 MHz, DMSO‐d6) *δ* 167.85, 145.81, 143.58, 141.28, 140.77, 140.12, 137.82, 136.44, 134.07, 131.83, 131.72, 130.51, 129.58, 128.56, 128.56, 128.17, 127.78, 127.55, 127.26, 126.34, 123.41, 121.39, 120.76, 120.58, 119.58, 118.92, 118.03, 117.35, 114.42, 112.37, 111.32, 65.83, 47.77, 36.11, 32.06, 31.93, 25.08, 24.33, and 24.27. **MS (ESI) of [C**_**53**_ **H**_**44**_ **ClN**_**6**_ **O**_**2**_ **+H]** ^**+**^ **(m/z)**: 858.7 Calcd: 857.5. **IR ( cm** ^**−1**^) 3434.03, 2936.11, 2249.74, 2123.86, 1772.27, 1654.50, 1493.34, 1234.12, 1029.89, 824.15, 762.74, and 623.20.

##### N‐(2‐(tert‐Butylamino)‐2‐Oxo‐1‐(1‐Phenyl‐9H‐Pyrido[3,4‐b]indol‐3‐Yl)ethyl)‐N‐(4‐Chlorophenyl)‐4‐(di(1H‐Indol‐3‐Yl)methyl)benzamide (12d)

Light orange solid, Yield (85%), m.p 177–179 °C. ^**1**^ **H NMR** (500 MHz, DMSO‐d6) *δ* 11.52 (s, 1H), 10.84 (d, *J* = 2.0 Hz, 2H), 8.25–8.18 (m, 2H), 8.03 (s, 1H), 8.00–7.96 (m, 2H), 7.65 (ddd, *J* = 15.3, 8.5, 4.9 Hz, 3H), 7.61–7.53 (m, 2H), 7.38 (d, *J* = 8.1 Hz, 2H), 7.32–7.21 (m, 7H), 7.17 (d, *J* = 7.9 Hz, 2H), 7.09–7.05 (m, 2H), 7.03 (d, *J* = 8.9 Hz, 2H), 6.92–6.87 (m, 2H), 6.75 (d, *J* = 2.1 Hz, 2H), 6.66 (s, 1H), 5.79 (s, 1H), and 1.30 (s, 9H). ^**13**^ **C NMR** (126 MHz, DMSO‐d6) *δ* 169.76, 168.10, 145.81, 145.17, 143.97, 141.31, 140.67, 140.27, 137.84, 136.46, 134.12, 131.75, 131.68, 130.44, 129.71, 129.61, 128.59, 128.47, 128.17, 127.85, 127.57, 127.28, 126.36, 125.69, 123.43, 121.32, 120.78, 120.56, 119.58, 118.94, 118.05, 117.37, 114.17, 112.40, 111.34, 50.30, and 28.32. **MS (ESI) of [C**_**53**_ **H**_**43**_ **ClN**_**6**_ **O**_**2**_ **+H]** ^**+**^ **(m/z)**: 831.9Calcd: 830.3. **IR ( cm** ^**−1**^)3432.07, 2250.01, 2124.01, 1771.99, 1658.21, 1376.11, 1230.75, 1027.07, 824.04, 762.80, and 623.02.

##### N‐(2‐(cyclohexylamino)‐2‐Oxo‐1‐(1‐Phenyl‐9H‐Pyrido[3,4‐b]indol‐3‐Yl)ethyl)‐4‐(di(1H‐Indol‐3‐Yl)methyl)‐N‐(4‐Methoxyphenyl)benzamide (12e)

Light red solid, Yield (92%), m.p 180–182 °C. ^**1**^ **H NMR** (500 MHz, DMSO‐d6) *δ* 11.46 (s, 1H), 10.79 (d, *J* = 2.0 Hz, 2H), 8.30 (d, *J* = 7.7 Hz, 1H), 8.19 (d, *J* = 7.9 Hz, 1H), 7.94 (dd, *J* = 6.4, 5.0 Hz, 3H), 7.63–7.58 (m, 3H), 7.55–7.49 (m, 2H), 7.33 (dd, *J* = 8.1, 0.5 Hz, 2H), 7.24 (dd, *J* = 11.0, 3.9 Hz, 1H), 7.20–7.11 (m, 6H), 7.09–6.99 (m, 4H), 6.83 (dd, *J* = 7.9, 7.1 Hz, 2H), 6.69 (s, 2H), 6.57 (s, 1H), 6.47 (d, *J* = 8.8 Hz, 2H), 5.73 (s, 1H), 3.72 (ddd, *J* = 13.6, 12.7, 3.6 Hz, 1H), 3.49 (s, 3H), 1.78 (d, *J* = 9.5 Hz, 1H), 1.70 (d, *J* = 11.3 Hz, 1H), 1.60 (d, *J* = 30.2 Hz, 2H), 1.50 (d, *J* = 12.7 Hz, 1H), 1.26 (s, 2H), and 1.15–1.02 (m, 3H). ^**13**^ **C NMR** (126 MHz, DMSO‐d6) *δ* 169.96, 167.98, 157.14, 145.43, 144.05, 141.26, 140.63, 137.92, 136.42, 134.49, 133.80, 131.73, 131.33, 129.56, 128.54, 128.42, 128.20, 128.11, 127.75, 127.35, 126.36, 123.38, 121.35, 120.73, 120.62, 119.52, 118.93, 118.01, 117.46, 114.28, 112.51, 112.33, 111.30, 66.01, 59.62, 54.87, 47.64, 32.04, 31.95, 25.09, and 24.29. **MS (ESI) of [C**_**56**_ **H**_**48**_ **N**_**6**_ **O**_**3**_ **+H]** ^**+**^ **(m/z)**: 853.3 Calcd: 852.4. **IR ( cm** ^**−1**^) 3434.63, 2934.18, 2250.09, 2124.14, 1659.15, 1152.54, 1242.90, 1026.98, 823.88, and 1762.80,623.27.

##### N‐(2‐(tert‐Butylamino)‐2‐Oxo‐1‐(1‐Phenyl‐9H‐Pyrido[3,4‐b]indol‐3‐Yl)ethyl)‐4‐(di(1H‐Indol‐3‐Yl)methyl)‐N‐(4‐Methoxyphenyl)benzamide (12f)

Pink solid, Yield (88%), m.p 185–187 °C. ^**1**^ **H NMR** (500 MHz, DMSO‐d6) *δ* 11.47 (s, 1H), 10.79 (d, *J* = 1.9 Hz, 2H), 8.19–8.14 (m, 2H), 7.97–7.95 (m, 2H), 7.94 (s, 1H), 7.60 (dd, *J* = 13.4, 7.9 Hz, 4H), 7.55–7.50 (m, 2H), 7.32 (d, *J* = 8.1 Hz, 2H), 7.24 (t, *J* = 7.4 Hz, 1H), 7.20–7.12 (m, 7H), 7.02 (t, *J* = 7.5 Hz, 2H), 6.83 (t, *J* = 7.5 Hz, 2H), 6.69 (s, 2H), 6.53 (s, 1H), 6.48 (d, *J* = 8.9 Hz, 2H), 5.73 (s, 1H), 3.49 (s, 3H), and 1.25 (s, 9H). ^**13**^ **C NMR** (126 MHz, DMSO‐d6) *δ* 168.19, 157.13, 151.10, 145.44, 144.43, 141.28, 140.49, 137.91, 136.42, 134.51, 133.96, 131.68, 131.24, 129.60, 128.55, 128.43, 128.17, 127.81, 127.35, 126.36, 123.39, 121.29, 120.73, 120.60, 119.52, 118.93, 118.02, 117.46, 114.03, 112.52, 112.35, 111.30, 110.23, 54.84, 50.19, 28.33, and 24.16. **MS (ESI) of [C**_**54**_ **H**_**46**_ **N**_**6**_ **O**_**3**_ **+H]** ^**+**^ **(m/z)**: 827.9Calcd: 826.4. **IR ( cm** ^**−1**^) 3431.80, 2249.75, 2123.80, 1659.27, 1509.86, 1378.47, 1237.33, 1026.81, 824.09, 762.42, and 622.99.

##### N‐(2‐(cyclohexylamino)‐2‐Oxo‐1‐(1‐Phenyl‐9H‐Pyrido[3,4‐b]indol‐3‐Yl)ethyl)‐4‐(di(1H‐Indol‐3‐Yl)methyl)‐N‐(4‐(trifluoromethyl)phenyl)benzamide (12g)

Light Pink solid, Yield (94%), m.p 154–156 °C. ^**1**^ **H NMR** (500 MHz, DMSO‐d6) *δ* 11.49 (s, 1H), 10.79 (s, 2H), 8.33 (d, *J* = 7.8 Hz, 1H), 8.22 (d, *J* = 7.8 Hz, 1H), 8.05 (s, 1H), 7.84 (d, *J* = 7.3 Hz, 2H), 7.61 (d, *J* = 8.2 Hz, 1H), 7.56 (dd, *J* = 13.0, 5.5 Hz, 3H), 7.50 (dd, *J* = 14.4, 7.1 Hz, 2H), 7.37–7.28 (m, 5H), 7.22 (d, *J* = 8.1 Hz, 3H), 7.18 (d, *J* = 8.1 Hz, 2H), 7.10 (d, *J* = 7.5 Hz, 2H), 7.02 (t, *J* = 7.5 Hz, 2H), 6.81 (t, *J* = 7.3 Hz, 2H), 6.69 (s, 3H), 5.74 (s, 1H), 3.85—3.50 (m, 1H), 1.77 (d, *J* = 10.1 Hz, 1H), 1.59 (d, *J* = 9.7 Hz, 3H), 1.49 (d, *J* = 11.4 Hz, 1H), 1.23 (d, *J* = 10.8 Hz, 2H), and 1.08 (dd, *J* = 24.3, 10.1 Hz, 3H). ^**13**^ **C NMR** (126 MHz, DMSO‐d6) *δ* 169.92, 167.58, 146.10, 145.10, 143.43, 141.31, 140.77, 136.44, 133.81, 131.78, 130.55, 129.65, 128.51, 128.14, 127.89, 127.62, 126.31, 124.32, 123.38, 122.71, 121.44, 120.74, 120.58, 119.57, 119.53, 118.86, 117.97, 117.29, 116.59, 114.48, 112.35, 111.32, 47.75, 36.10, 31.99, 31.85, 25.05, 24.25, 24.16, and23.57. **MS (ESI) of [C**_**56**_ **H**_**45**_ **F**_**3**_ **N**_**6**_ **O**_**2**_ **+H]** ^**+**^ **(m/z)**: 891.4 Calcd: 890.4. **IR ( cm** ^**−1**^) 3430.90, 2250.73, 2125.03, 1658.68, 1377.94, 1233.33, 1025.75, 824.44, 763.41, and 624.59.

##### N‐(2‐Tert‐Butylamino)2‐Oxo‐1‐(1‐Phenyl‐9H‐Pyrido[3,4‐b]indol‐3‐Yl)ethyl‐4‐(di(1H‐Indol‐3‐Yl)methyl)‐N‐(4‐(trifluoromethyl)phenyl)benzamide (12Hr)

Light Pink solid, Yield (95%), m.p 158–160 °C. ^**1**^ **H NMR** (500 MHz, DMSO‐d6) *δ* 11.53 (s, 1H), 10.83 (d, *J* = 1.8 Hz, 2H), 8.25 (d, *J* = 9.2 Hz, 2H), 8.08 (s, 1H), 7.94—7.91 (m, 2H), 7.66 (d, *J* = 8.2 Hz, 1H), 7.64—7.57 (m, 3H), 7.56—7.52 (m, 1H), 7.45 (d, *J* = 8.1 Hz, 2H), 7.39—7.34 (m, 4H), 7.32—7.26 (m, 3H), 7.23 (d, *J* = 8.3 Hz, 2H), 7.15 (d, *J* = 8.0 Hz, 2H), 7.06 (t, *J* = 7.6 Hz, 2H), 6.86 (dd, *J* = 7.9, 7.1 Hz, 2H), 6.74 (d, *J* = 2.1 Hz, 2H), 6.69 (s, 1H), 5.79 (s, 1H), and 1.27 (s, 9H). ^**13**^ **C NMR** (126 MHz, DMSO‐d6) *δ* 169.88, 167.86, 146.13, 145.26, 143.85, 141.37, 140.73, 137.83, 136.49, 133.90, 131.78, 130.51, 130.06, 129.71, 128.60, 128.53, 128.28, 128.16, 127.98, 127.67, 126.36, 124.93, 124.40, 124.37, 123.43, 122.76, 121.41, 120.82, 120.60, 118.92, 118.05, 117.35, 114.23, 112.42, 111.38, 66.25, 50.35, and 28.29.**MS (ESI) of [C**_**54**_ **H**_**43**_ **F**_**3**_ **N**_**6**_ **O**_**2**_ **+H]** ^**+**^ **(m/z)**: 865.5 Calcd: 864.3. **IR ( cm** ^**−1**^) 3430.95, 2250.55, 2124.40, 1659.17, 1380, 1228.34, 1026.12, 824.22, 763.37, and 624.35.

##### N‐(2‐(cyclohexylamino)‐2‐Oxo‐1‐(1‐Phenyl‐9H‐Pyrido[3,4‐b]indol‐3‐Yl)ethyl)‐4‐(di(1H‐Indol‐3‐Yl)methyl)‐N‐(p‐Tolyl)benzamide (12i)

Light Orange solid, Yield (85%), m.p 176–178 ^*°*^C. ^**1**^ **H NMR** (500 MHz, DMSO) *δ* 11.45 (s, 1H), 10.78 (d, *J* = 2.1 Hz, 2H), 8.31 (d, *J* = 7.9 Hz, 1H), 8.18 (d, *J* = 7.9 Hz, 1H), 7.95 (s, 1H), 7.93–7.88 (m, 2H), 7.61–7.57 (m, 3H), 7.54–7.48 (m, 2H), 7.31 (d, *J* = 8.1 Hz, 2H), 7.24–7.21 (m, 1H), 7.17 (d, *J* = 8.2 Hz, 2H), 7.15–7.10 (m, 4H), 7.03–6.99 (m, 3H), 6.84–6.80 (m, 2H), 6.71 (d, *J* = 8.3 Hz, 2H), 6.65 (d, *J* = 1.8 Hz, 2H), 6.57 (s, 1H), 5.71 (s, 1H), 3.75–3.67 (m, 1H), 2.00 (s, 3H), 1.78 (d, *J* = 9.7 Hz, 1H), 1.68 (dd, *J* = 12.5, 2.9 Hz, 1H), 1.63–1.56 (m, 2H), 1.49 (dd, *J* = 8.8, 3.9 Hz, 1H), 1.24 (d, *J* = 12.1 Hz, 2H), and 1.15–1.03 (m, 3H). ^**13**^ **C NMR** (126 MHz, DMSO) *δ* 169.97, 167.91, 145.47, 144.02, 141.25, 140.58, 137.89, 136.42, 135.18, 134.47, 134.44, 131.70, 129.86, 129.57, 128.54, 128.43, 128.18, 128.13, 127.88, 127.76, 127.33, 126.36, 123.39, 121.39, 120.73, 120.62, 119.53, 118.92, 118.00, 117.43, 114.20, 112.32, 111.29, 66.04, 47.65, 32.03, 25.09, 24.28, 24.23, and 20.27. **MS (ESI) of [C**_**56**_ **H**_**48**_ **H**_**6**_ **O**_**2**_ **+H]** ^**+**^ **(m/z)**: 837.4Calcd: 836.4. **IR ( cm** ^**−1**^) 3276, 2924, 2850, 1048, 1512, 1320, 1246, 1020, 738, and 626.

##### N‐(2‐(tert‐Butylamino)‐2‐Oxo‐1‐(1‐Phenyl‐9H‐Pyrido[3,4‐b]indol‐3‐Yl)ethyl)‐4‐(di(1H‐Indol‐3‐Yl)methyl)‐N‐(p‐Tolyl)benzamide (12j)

Light Pink solid, Yield (82%), m.p 172–174 °C. ^**1**^ **H NMR** (500 MHz, CDCl_3_) *δ* 9.04 (s, 1H), 8.72 (s, 1H), 8.19 (d, *J* = 16.2 Hz, 2H), 8.09 (s, 1H), 8.03 (d, *J* = 7.8 Hz, 1H), 7.80 (d, *J* = 7.3 Hz, 2H), 7.49 (dd, *J* = 10.5, 4.5 Hz, 4H), 7.41 (t, *J* = 7.4 Hz, 1H), 7.28–7.25 (m, 2H), 7.23 (d, *J* = 4.6 Hz, 1H), 7.14–7.10 (m, 4H), 7.06 (d, *J* = 7.1 Hz, 2H), 6.93 (dd, *J* = 8.1, 2.8 Hz, 4H), 6.89 (dd, *J* = 7.1, 2.1 Hz, 2H), 6.81 (d, *J* = 8.1 Hz, 2H), 6.42 (s, 1H), 6.25 (s, 1H), 6.19 (s, 1H), 5.62 (s, 1H), 2.18 (s, 3H), and 1.26 (s, 9H). ^**13**^ **C NMR** (126 MHz, CDCl_3_) *δ* 171.66, 167.63, 145.44, 141.01, 140.80, 139.69, 138.00, 136.64, 136.60, 133.80, 132.54, 131.23, 129.20, 129.02, 128.93, 128.78, 128.49, 128.09, 127.92, 126.84, 126.82, 123.82, 121.95, 121.70, 121.63, 120.35, 119.68, 119.63, 118.86, 118.69, 114.39, 111.78, 111.18, 102.46, 67.23, 51.13, 39.88, and 28.60. **MS (ESI) of [C**_**54**_ **H**_**46**_ **N**_**6**_ **O**_**2**_ **+H]** ^**+**^ **(m/z)**: 811.5Calcd: 810.4. **IR (cm** ^**−1**^)3286, 2960, 2860, 1650, 1510, 1323, 1238, 1010, 741, and 621.

##### N‐(2‐(cyclohexylamino)‐2‐Oxo‐1‐(1‐Phenyl‐9H‐Pyrido[3,4‐b]indol‐3‐Yl)ethyl)‐4‐(di(1H‐Indol‐3‐Yl)methyl)‐N‐(4‐Nitrophenyl)benzamide (12k)

Pink solid, Yield (88%), m.p 174–176 °C. ^**1**^ **H NMR** (500 MHz, DMSO) *δ* 11.47 (s, 1H), 10.79 (s, 2H), 8.39 (d, *J* = 7.8 Hz, 1H), 8.20 (d, *J* = 7.9 Hz, 1H), 8.04 (s, 1H), 7.87–7.85 (m, 2H), 7.77 (d, *J* = 9.2 Hz, 2H), 7.61–7.57 (m, 3H), 7.56–7.50 (m, 5H), 7.42 (d, *J* = 8.3 Hz, 2H), 7.31 (d, *J* = 8.1 Hz, 2H), 7.23 (d, *J* = 6.4 Hz, 2H), 7.18 (d, *J* = 8.3 Hz, 2H), 7.08 (d, *J* = 8.0 Hz, 2H), 7.02—6.98 (m, 2H), 6.76 (s, 1H), 6.71 (t, *J* = 2.2 Hz, 2H), 5.73 (s, 1H), 3.75—3.69 (m, 1H), 1.83 (d, *J* = 10.0 Hz, 1H), 1.62 (d, *J* = 11.1 Hz, 3H), 1.52 (s, 1H), 1.23 (s, 2H), and1.12 (d, *J* = 11.4 Hz, 3H). ^**13**^ **C NMR** (126 MHz, DMSO‐d6) *δ* 167.85, 145.81, 143.58, 141.28, 140.77, 140.12, 137.82, 136.44, 134.07, 131.83, 131.72, 130.51, 129.58, 128.56, 128.56, 128.17, 127.78, 127.55, 127.26, 126.34, 123.41, 121.39, 120.76, 120.58, 119.58, 118.92, 118.03, 117.35, 114.42, 112.37, 111.32, 65.83, 47.77, 36.11, 32.06, 31.93, 25.08, 24.33, and 24.27. **MS (ESI) of [C**_**53**_ **H**_**45**_ **N**_**7**_ **O**_**4**_ **+H]** ^**+**^ **(m/z)**: 868.3Calcd: 867.4. **IR  cm** ^**−1**^) 3297, 2934, 2849, 1660, 1519, 1327, 1242, 1010, 738, and 626.

##### N‐(2‐(tert‐Butylamino)‐2‐Oxo‐1‐(1‐Phenyl‐9H‐Pyrido[3,4‐b]indol‐3‐Yl)ethyl)‐4‐(di(1H‐Indol‐3‐Yl)methyl)‐N‐(4‐Nitrophenyl)benzamide (12l)

Pink solid, Yield (78%), m.p 184–186 °C. ^**1**^ **H NMR** (500 MHz, CDCl_3_) *δ* 8.74 (s, 1H), 8.15 (s, 1H), 8.14 (s, 1H), 8.06 (dd, *J* = 10.9, 4.8 Hz, 2H), 8.00 (d, *J* = 1.6 Hz, 1H), 7.83 (dd, *J* = 9.8, 8.1 Hz, 4H), 7.57–7.52 (m, 4H), 7.50–7.47 (m, 2H), 7.32–7.27 (m, 4H), 7.19 (t, *J* = 8.9 Hz, 4H), 7.14—7.10 (m, 2H), 7.07 (d, *J* = 8.2 Hz, 2H), 6.94 (dd, *J* = 7.8, 7.2 Hz, 2H), 6.58 (s, 1H), 6.38 (d, *J* = 1.6 Hz, 1H), 6.35 (d, *J* = 1.6 Hz, 1H), 5.72 (s, 1H), 1.30 (s, 9H). ^**13**^ **C NMR** (126 MHz, CDCl_3_) *δ* 171.29, 167.28, 148.67, 146.53, 145.45, 144.60, 141.48, 140.89, 137.75, 136.65, 136.63, 133.19, 132.61, 131.25, 129.81, 129.26, 129.10, 128.70, 128.44, 127.91, 126.76, 123.63, 123.50, 122.00, 121.63, 120.71, 119.69, 119.65, 119.16, 118.65, 114.55, 111.74, 111.15, 66.75, 51.53, 40.00, and 28.63. **MS (ESI) of [C**_**53**_ **H**_**43**_ **N**_**7**_ **O**_**4**_ **+H]** ^**+**^ **(m/z)**: 842.2Calcd: 841.3. **IR ( cm** ^**−1**^). 3314, 2978, 2898, 1685, 1522, 1326, 742, 856, and 600.

##### Evaluating the Antibacterial Potential of Hybrids against Gram‐Negative and Gram‐Positive Bacteria

To assess the in vitro antibacterial activity of the synthesized hybrid compounds, an agar‐diffusion assay was employed using the Kirby–Bauer method against Gram‐positive and Gram‐negative bacteria. Two Gram‐positive strains, *Staphylococcus aureus, Bacillus subtilis,* and two Gram‐negative strains, *Pseudomonas aeruginosa, and Acinetobacter baumannii*, were used to screen the antibacterial activity of the hybrids. Briefly, 100 μl of the bacterial cultures in their active phase of growth (containing 1 × 10^8^ CFU mL^−^^1^) were swabbed onto Mueller–Hinton agar plates. Subsequently, the freshly seeded plates were punched with five wells each of 8 mm diameter using a sterile borer. The first two wells were then filled with 100 μl of reference/parent compounds at a concentration of 5 mg mL^−1^. To the third well, 100 μl of hybrid compound (5 mg mL^−1^) was added. 100 μl of 50 μg mL^−1^ gentamicin (standard drug) and 100 μl of DMSO (solvent control) were added to the fourth and fifth wells, respectively. After that, the agar plates were incubated for 18 h at 37 °C, and the zones of inhibition (in mm) were measured the next day. To achieve statistically significant results, each hybrid was examined three times.

##### Determining the Minimum Inhibitory Concentrations (MIC) of the Hybrids

Previously established protocols were used to determine the MIC of the synthesized hybrids for the selected Gram‐positive and Gram‐negative bacterial strains using the standard 96‐well microtiter broth dilution method. In each well, 100 μl of double‐strength Mueller‐Hinton broth was added, followed by dispensing 100 μl of hybrid compound (5 mg mL) in the first well. The compounds were then serially diluted (1:1) by transferring 100 μl suspension from the previous well to the next one, repeating this process until reaching the desired lowest dilutions of the compounds. To each well, barring the negative controls, 10 μl of bacterial inoculum was added. Negative control wells consisted of only Mueller‐Hinton broth, while positive growth controls included wells with inoculated Mueller‐Hinton broth devoid of hybrid compounds. The MIC plates were incubated at 37 °C for 18–20 h. The next day, 20 μl of resazurin dye (working concentration 0.01%) was added to each well, followed by incubation at 37 °C for 2 h. Since resazurin is a redox dye, a visible color change from blue to pink was observed in the wells with microbial growth. However, the lowest concentration at which there was no visible color change after the addition of the dye was identified as the MIC, indicating complete inhibition of microbial growth.

##### Molecular Docking

Molecular docking was conducted using the Schrödinger suite (2022–1 release) with calculations based on the OPLS4 forcefield. First, the crystal structure of DNA gyrase (PDB ID: 6KZV) was downloaded from the PDB and used to dock its native ligand in the active site to determine the predictive ability of the docking method. This protein was then modified by adding missing hydrogens, side chains, and loops using Prime. Epik was used to generate tautomers and heteroatomic states at pH 7.0 ± 2.0, and water molecules were deleted 5 Å away from the binding cavity. The hydrogen network was optimized and minimized to an RMSD of 0.3 Å. Subsequently, the binding site was minimized using Prime. Compounds for docking were drawn, saved as ChemDraw files, imported into the workspace, and prepared using the LigPrep module to generate 3D models with the lowest energy conformation. The Glide‐XP protocol module was employed to dock these compounds into the active site of DNA gyrase; PDB ID: 6KZV. This process involved defining the ligand box by selecting a box using the centroid of selected residues with dimensions under 20 Å and performing conformational sampling within a 2.5 kcal mol^−1^ energy range.

##### DFT Calculation

The molecular structures of all the synthesized compounds were optimized using Gaussian 16 W software. The B3LYP/6‐ 311++G (d, p) basis set was employed to optimize the structure and further calculate the electronic properties of the potent compounds. The FMOs, HOMO, and LUMO were visualized using Gauss View 6.0 software to understand the effect of substituents on the reactivity of compounds.

## Supporting Information

The details of spectroscopic data ^1^H NMR, ^13^C NMR, IR, MS) and data associated with biological results for model compounds associated with this article can be found in the Supporting Information.

## Conflict of Interest

The authors declare no conflict of interest.

## Supporting information

Supplementary Material

## Data Availability

The data that support the findings of this study are available in the supplementary material of this article.
