# Supplementary material for: One‐Pot Synthesis of Novel β‐Carboline‐{α‐Acylaminoamide}‐Bisindole Derivatives: Antibacterial Evaluation, Molecular Docking, and Density Functional Theory Studies
Source: ChemistryOpen. 2025 Sep 17;14(12):e202500245. doi: 10.1002/open.202500245 (PMC12680560; doi:10.1002/open.202500245)
Supplement: Supplementary file 1 — Supplementary Material [file OPEN-14-e202500245-s001.pdf]

One-Pot Synthesis of Novel β-Carboline-{*α-acylaminoamide*}-Bisindole Derivatives: Antibacterial Evaluation, Molecular Docking and DFT studies

Ankit Kumar Atri[a], Lavanya Khullar[b], Gobind Kumar[c], Sahil Mishra[c], Tamanna Dua[a], Vinay Singh[a], Kusum Harjai[b], Parvesh Singh[c]*, and Vasundhara Singh[a]*

[a]*Department of Applied Sciences, Punjab Engineering College (Deemed to be University), Sector-12, Chandigarh, 160012, India.*

[b]*Department of Microbiology, Panjab University, Sector-25, Chandigarh, 160014, India.*

[c]*School of Chemistry and Physics, University of KwaZulu-Natal, P/Bag X54001, Westville, Durban 4000, South Africa*

**Corresponding Author E-mail: vasun7@yahoo.co.in, singhp4@ukzn.ac.za*

SUPPORTING INFORMATION

1. CHEMISTRY

A. General Information……………………………………..

B. Characterisation of compounds.

C. ^{1}H NMR, ^{13}C NMR and Mass spectra of Compound 6….S1-S3

D.^{1}H NMR, ^{13}C NMR and Mass spectra of Compound 9 …S4-S6

E.^{1}H NMR, ^{13}C NMR, Mass and FT-IR spectra of compound 12a…S7- S10

F. ^{1}H NMR, ^{13}C NMR, Mass and FT-IR spectra of Compound 12b…S11-S14

G. ^{1}H NMR, ^{13}C NMR, Mass and FT-IR spectra of compound 12c…S15-S18

H. ^{1}H NMR, ^{13}C NMR, Mass and FT-IR spectra of compound 12d…S19-S22

I.^{1}H NMR, ^{13}C NMR, Mass and FT-IR spectra of compound 12e…S23-S26

J. ^{1}H NMR, ^{13}C NMR, Mass and FT-IR spectra of compound 12f…S27-S30

K. ^{1}H NMR, ^{13}C NMR, Mass and FT-IR spectra of compound 12g…S31-S34

L. ^{1}H NMR, ^{13}C NMR, Mass and FT-IR spectra of compound 12h…S35-S38

M. ^{1}H NMR, ^{13}C NMR, Mass and FT-IR spectra of compound 12i…S39-S42

N. ^{1}H NMR, ^{13}C NMR, Mass and FT-IR spectra of compound 12j…S43-S46

O. ^{1}H NMR, ^{13}C NMR, Mass and FT-IR spectra of compound 12k…S47-S50

P. ^{1}H NMR, ^{13}C NMR, Mass and FT-IR spectra of compound 12l…S51-S54

2. BIOLOGY

A. Bacterial strains and growth conditions

B. AHL extraction

C. Statistical Analysis

D. In vitro antibacterial activity of the synthesized compounds 12a-l… S55

3. Molecular docking………S56

1. CHEMISTRY

A. General information

All the solvents are of analytical grade having purity (>99.9%) were used for all reactions. When necessary and specified in the protocols, solvents were purchased extra dry anhydrous from Aldrich or Alfa aesar companies. THF were distilled prior
to use and was stored over activated 4 Å molecular sieves and KOH respectively. All chemicals were obtained from commercial suppliers (Sigma Aldrich, Acros or Alfa-aesar) and were used as received, unless otherwise stated was synthesized according to previously published protocols.

CHROMATOGRAPHY:

All reactions and Column chromatography purifications were monitored by using Merck precoated Silica Gel 60 F ^{254} analytical Thin Layer Chromatography (TLC) plates of 0.25 mm thickness. The detection on TLC plates was performed by UV light at 254 or 365 nm or using a spray and heating the plates.

INSTRUMENTATION:

The ^{1}H NMR spectra were recorded on a Bruker AV-500 spectrophotometer at 500 MHz. ^{13}C NMR spectra was recorded on Bruker AV-500 spectrophotometer at 126 MHz using the residual non-deuterated solvent as reference. The chemical shifts (δ) and coupling constants (J) are expressed in ppm and Hertz respectively. Multiplicities were indicated as s (singlet), d (doublet), t (triplet), q (quadruplet) and m (multiplet). A broad signal is mentioned with br preceding the multiplicity.Mass spectra were recorded on in the service commune and were measured after calibration in ES-TOF experiments on a Bruker Daltonic micro tof mass spectrometer. The Infrared spectral data of the compounds were obtained using PerkinElmer spectrum version FTIR instrument

B. Characterisation of compounds

Methyl-1-phenyl-9H-pyrido[3,4-b]indole-3-carbaldehyde (6) :

White solid, Yield (75%), m.p 313-314°C. **^{1}H NMR** (500 MHz, DMSO-d6) δ 12.06 (s, 1H), 10.18 (s, 1H), 8.84 (s, 1H), 8.46 (d, *J* = 7.9 Hz, 1H), 8.09 (dt, *J* = 3.0, 1.7 Hz, 2H), 7.73(d, *J*=8.2Hz, 1H), 7.70 – 7.58 (m, 4H), 7.37 (td, *J* = 7.6, 0.9 Hz, 1H). **^{13}C NMR** (126 MHz, DMSO-d6) δ 192.94, 143.20, 142.51,

141.53, 137.21, 135.33, 129.20, 129.03, 128.81, 128.77, 128.49, 122.08, 121.29, 120.56, 113.75, 112.85. **MS (ESI) of [$C_{18}H_{12}N_2O$+Na]$^+$(m/z)**: 295.1; Calcd: 272.1.

4-(di(1H-indol-3-yl)methyl)benzoic acid (9):

Red solid, Yield (95%), m.p 248-250 ºC. **^{1}H NMR** (500 MHz, DMSO-d6) δ 12.68 (s, 1H), 10.87 (d, *J* = 2.0 Hz, 2H), 7.87 – 7.84 (m, 2H), 7.46 (d, *J* = 8.2 Hz, 2H), 7.36 (d, *J* = 8.1 Hz, 2H), 7.28 (d, *J* = 8.0 Hz, 2H), 7.06 – 7.02 (m, 2H), 6.89 – 6.85 (m, 4H), 5.92 (s, 1H). **^{13}C NMR** (126 MHz, DMSO-d6) δ 171.94, 167.39, 149.72, 136.46, 129.10, 128.22, 126.40, 123.56, 120.83, 118.87, 118.14, 117.25, 111.38, 59.63. **MS (ESI) of [$C_{24}H_{18}N_2O_2$+Na]$^+$ (m/z)**: 389.5; Calcd: 266.1.

S1. ^{1}H NMR Spectra of Compound 6 in DMSO-d6

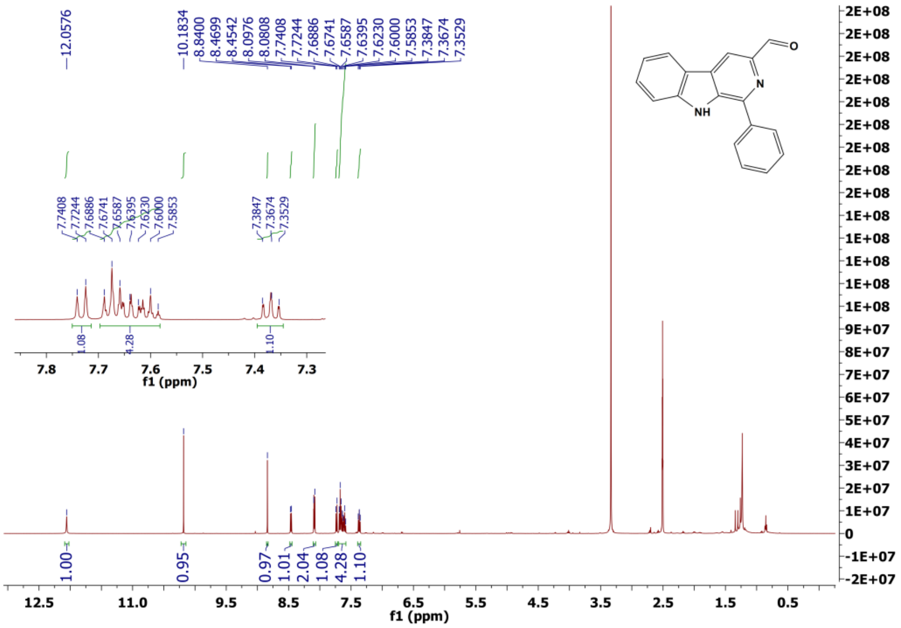

S2. ^{13}C NMR Spectra of Compound 6 in DMSO-d6

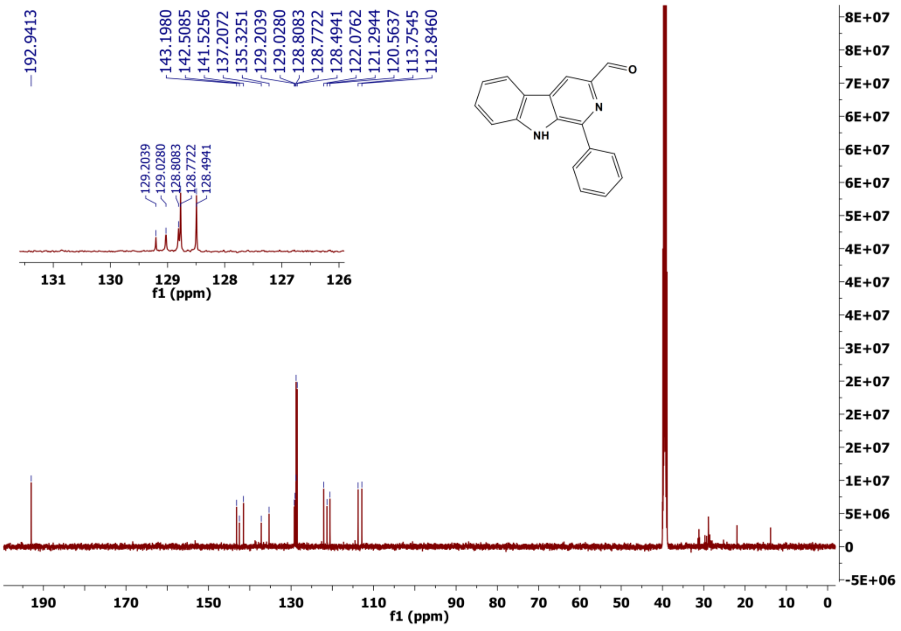

S3. Mass Spectra of Compound 6

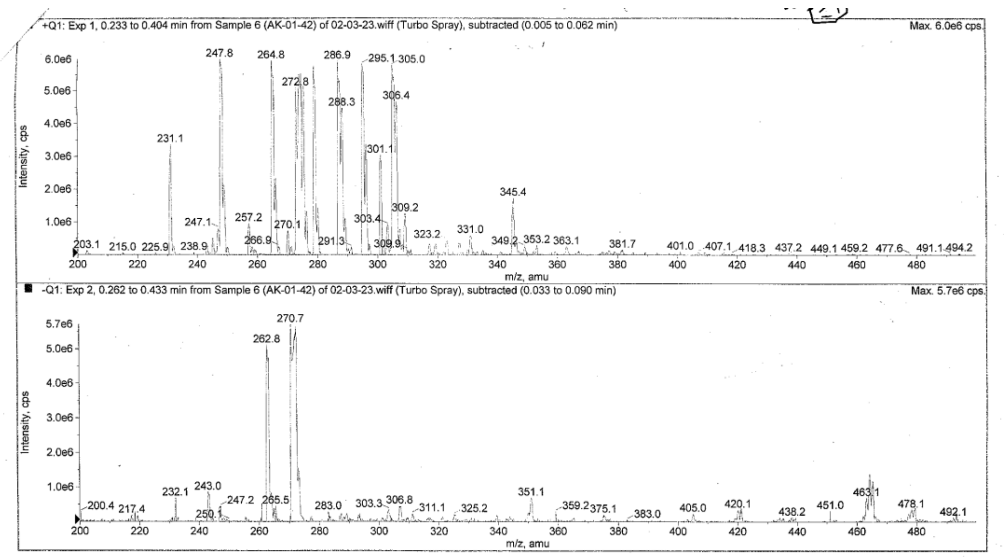

S4. ^{1}H NMR Spectra of Compound 9 in DMSO-d6

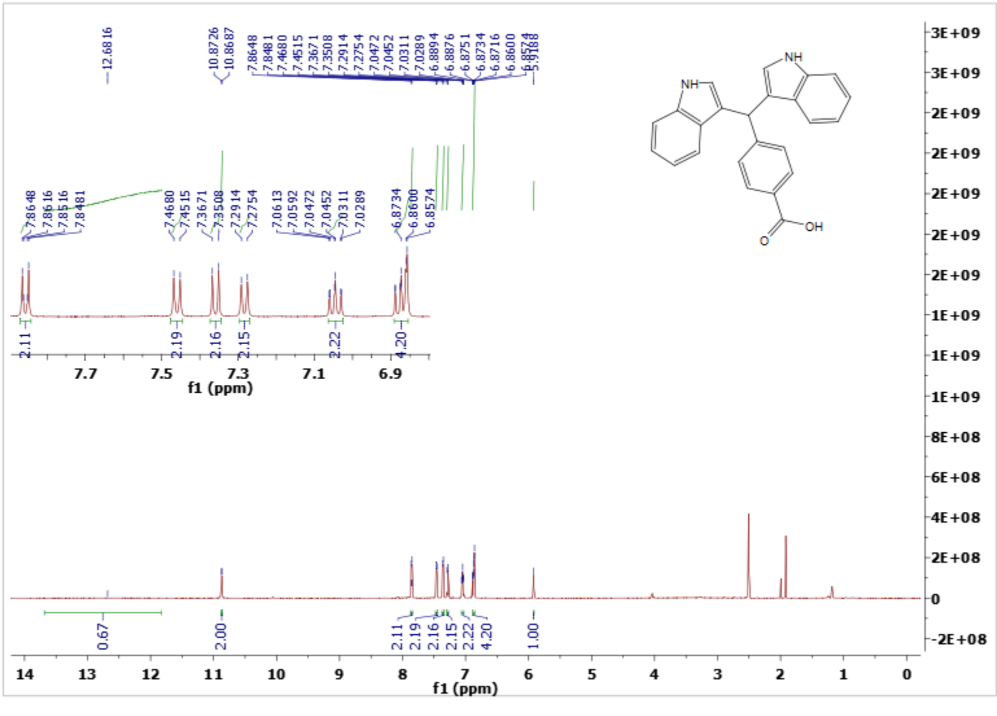

S5. ^{13}C NMR Spectra of Compound 9 in DMSO-d6

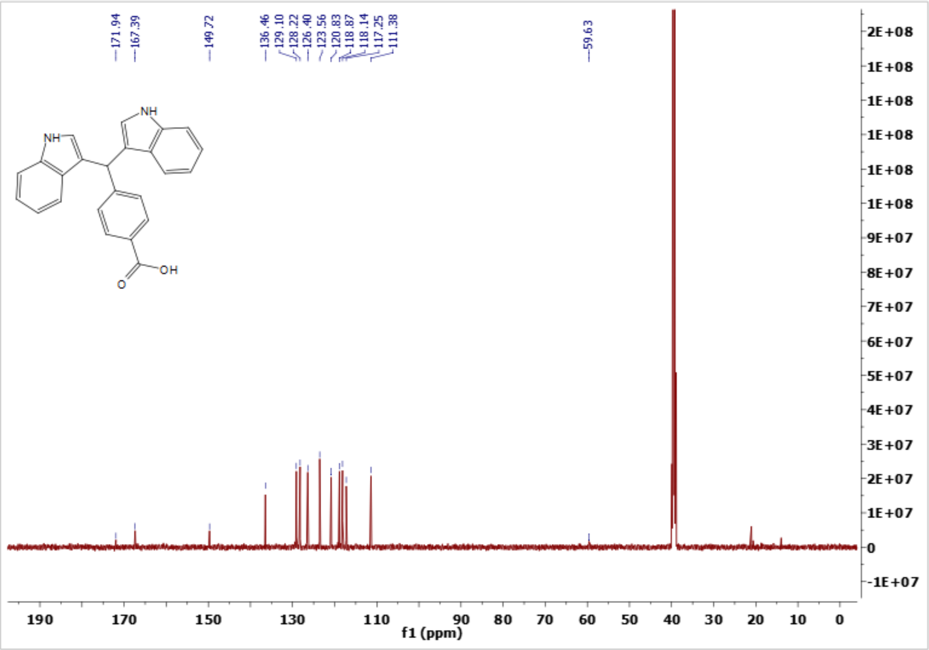

S6. Mass Spectra of Compound 9

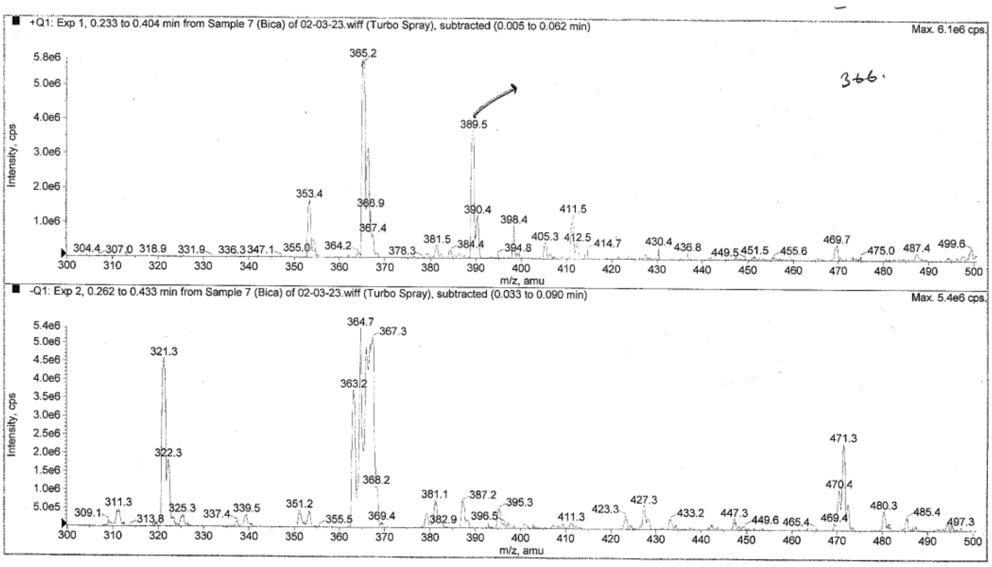

S7. ^{1}H NMR Spectra of Compound 12a in DMSO-d6

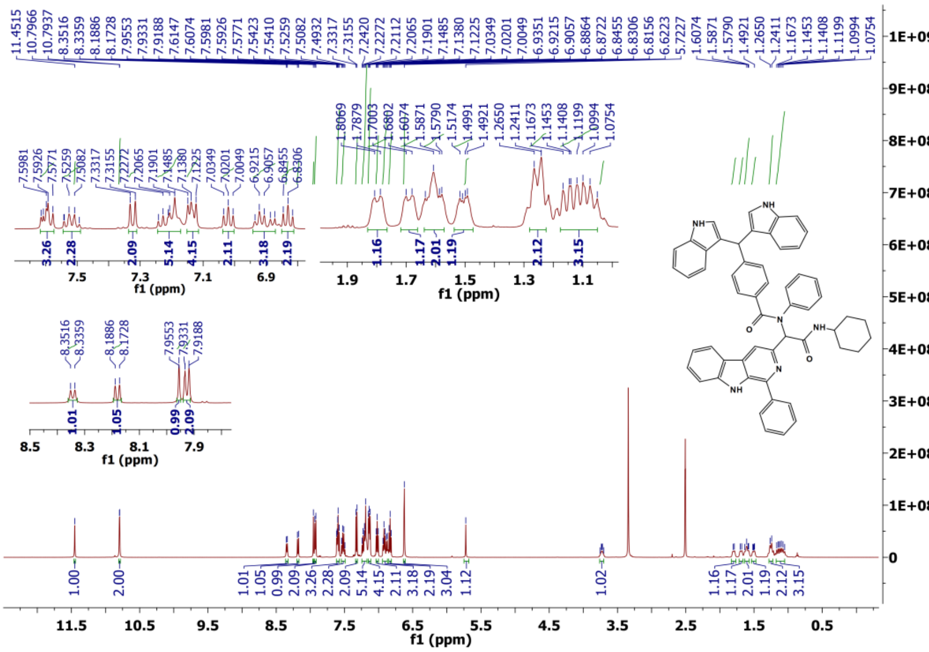

S8. ^{13}C NMR Spectra of Compound 12a in DMSO-d6

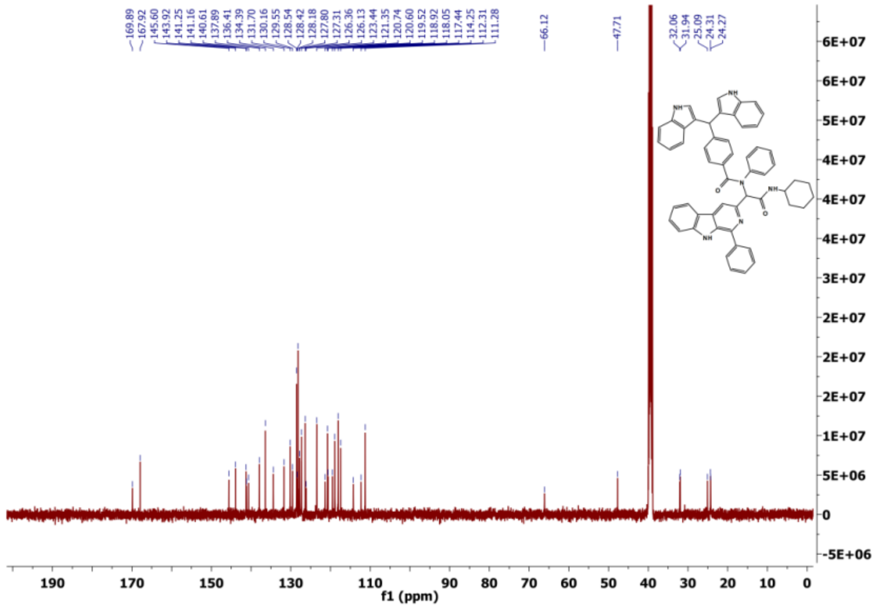

S9. Mass Spectra of Compound 12 a

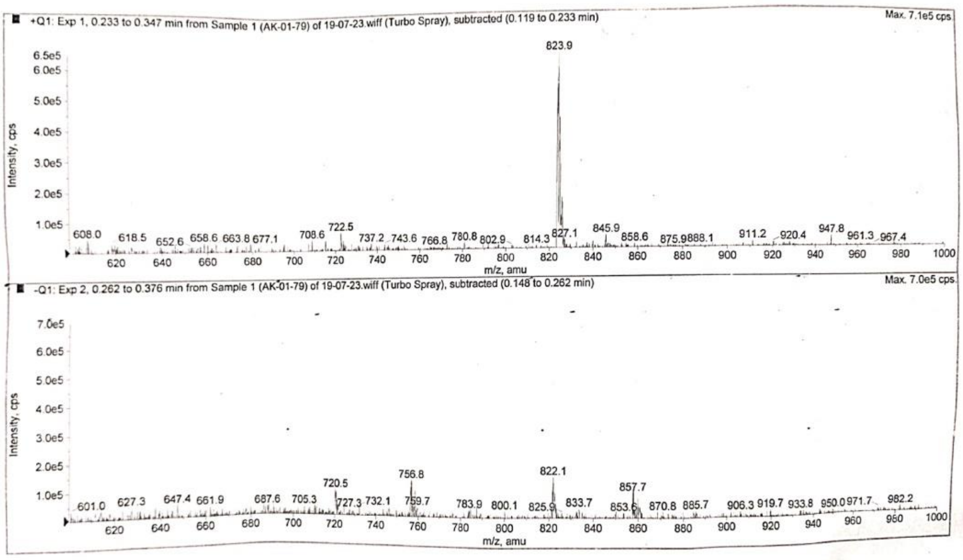

S10. FT-IR Spectra of Compound 12a

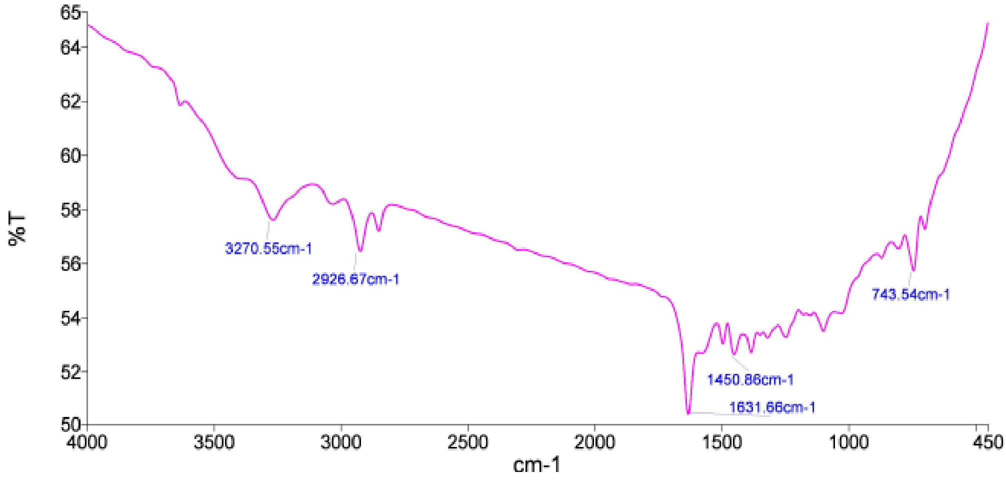

S11. ^{1}H NMR Spectra of Compound 12b in DMSO-d6

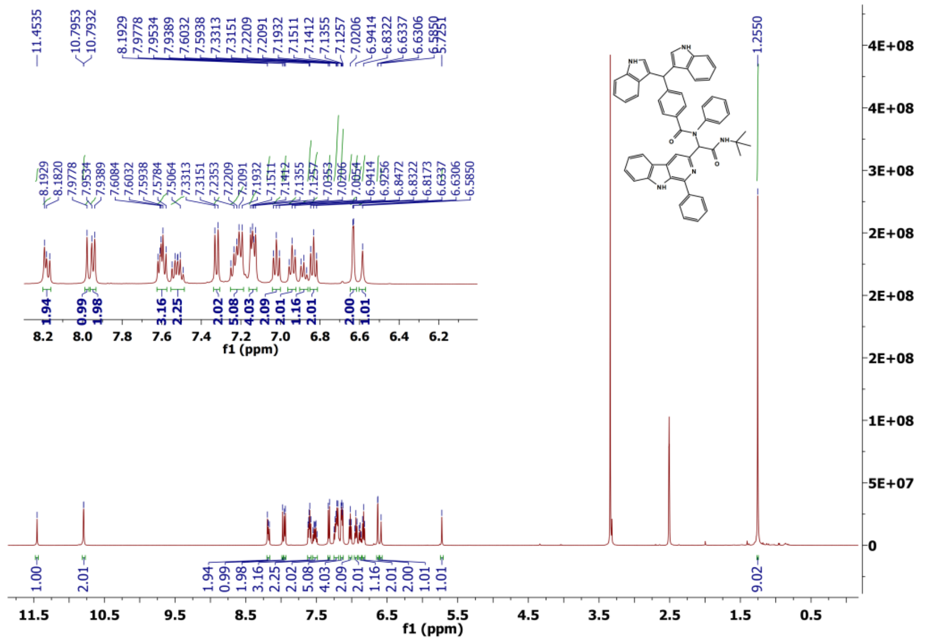

S12. ^{13}C NMR Spectra of Compound 12b in DMSO-d6

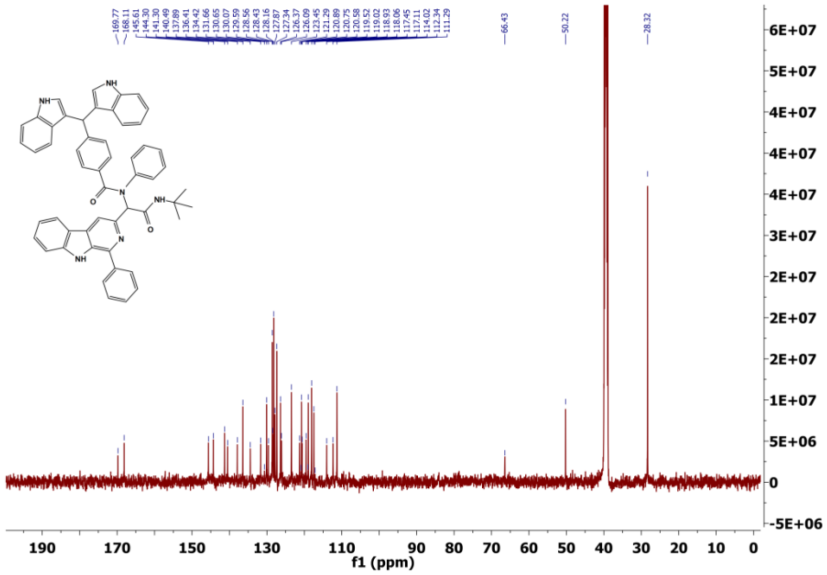

S13. Mass Spectra of Compound 12b

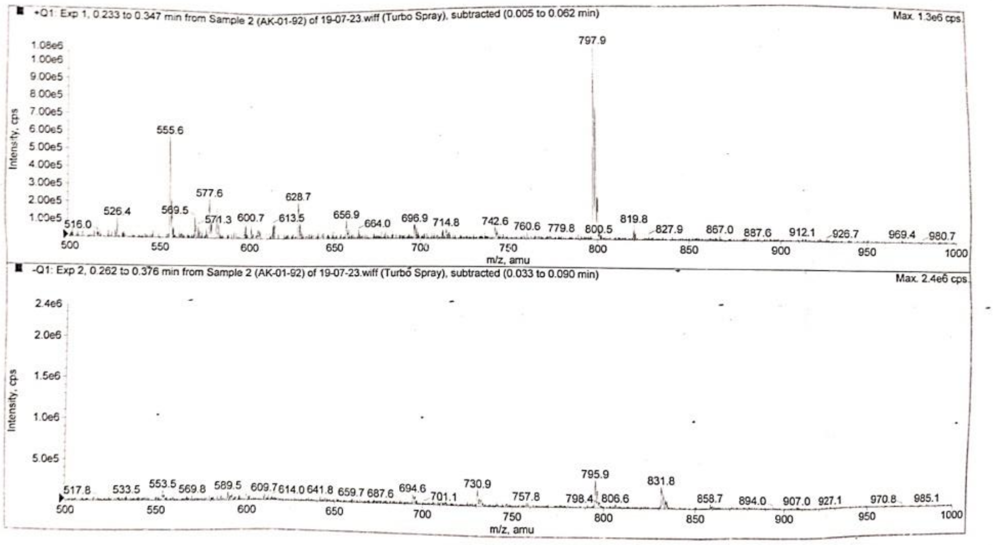

S14. FT-IR Spectra of Compound 12b

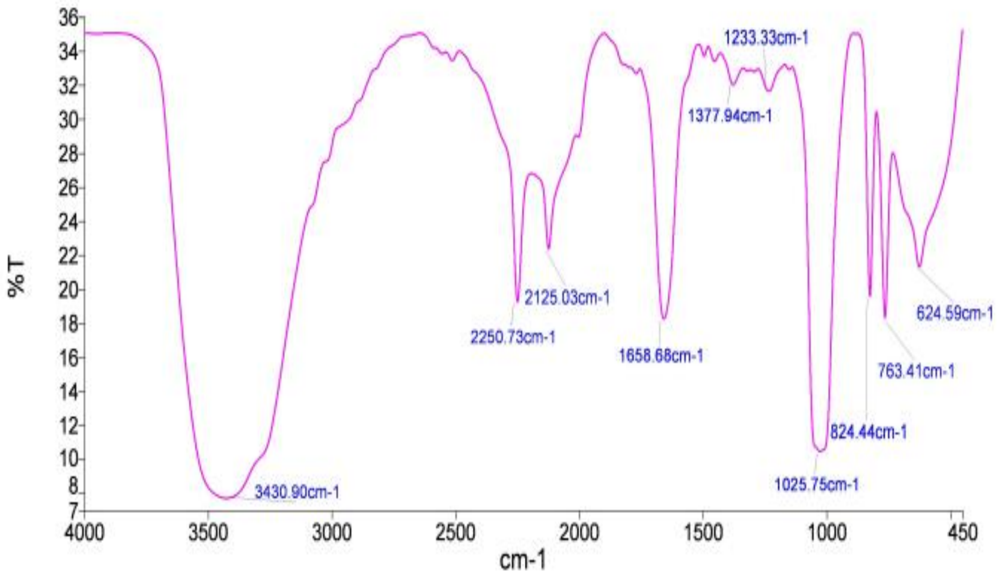

S15. ^{1}H NMR Spectra of Compound 12c in DMSO-d6

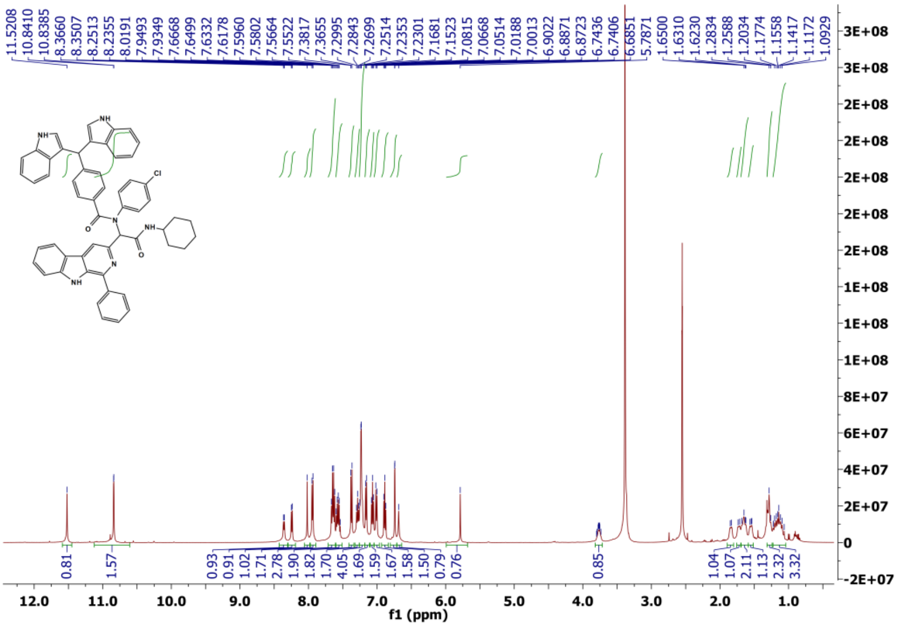

S16. ^{13}C NMR Spectra of Compound 12c in DMSO-d6

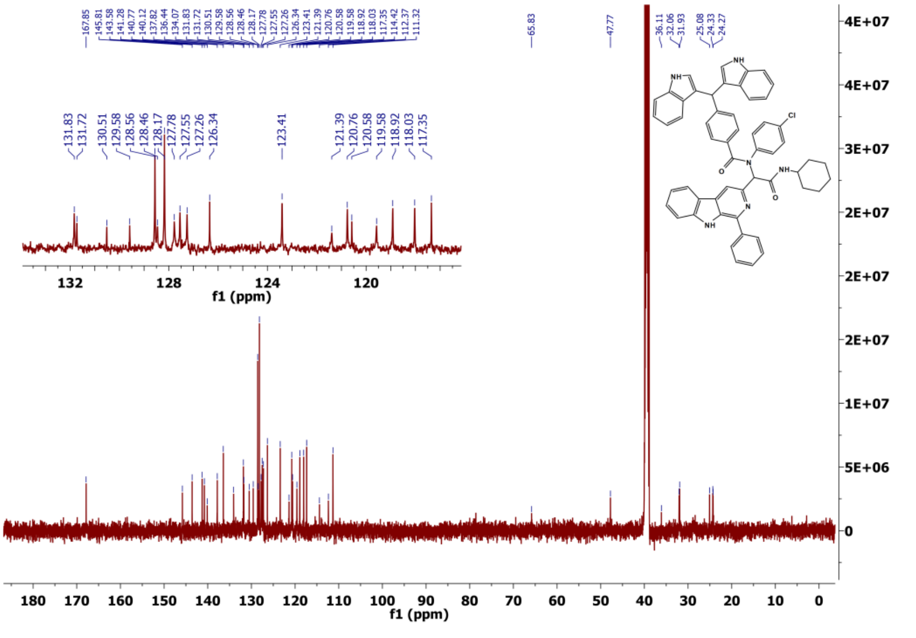

S17. Mass Spectra of Compound 12d

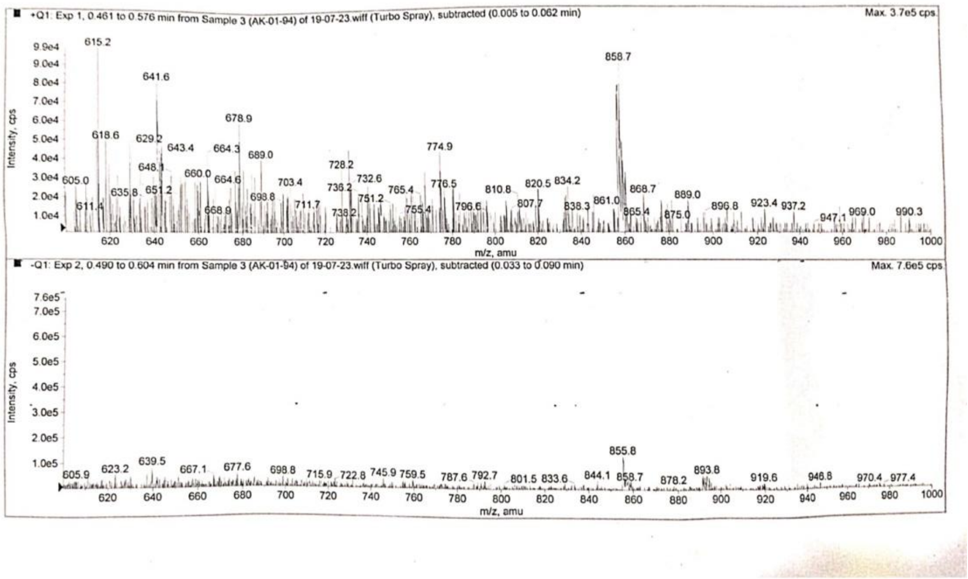

S18. FT-IR Spectra of Compound 12c

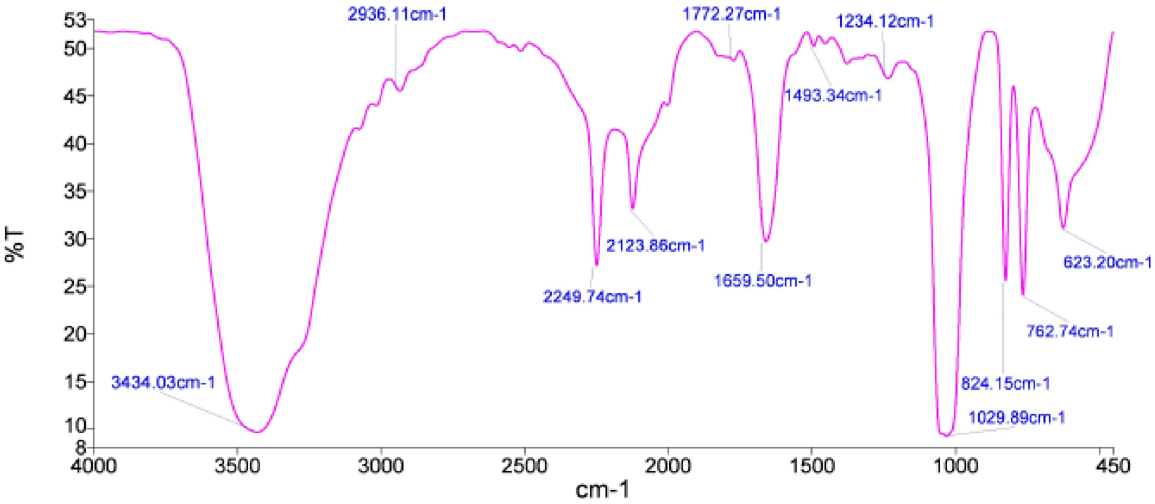

S19. ^{1}H NMR Spectra of Compound 12d in DMSO-d6

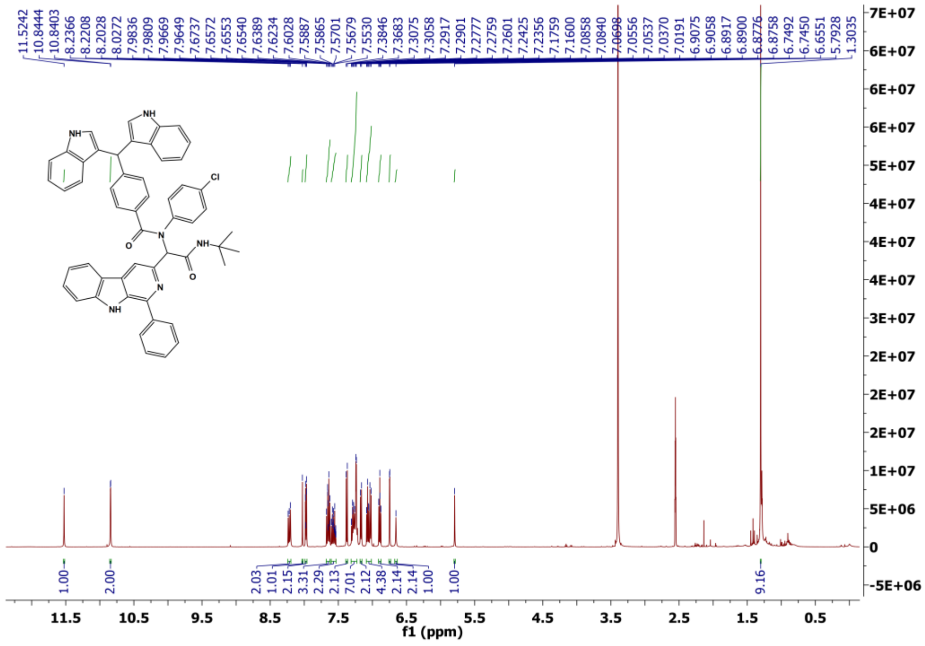

S20. ^{13}C NMR Spectra of Compound 12d in DMSO-d6

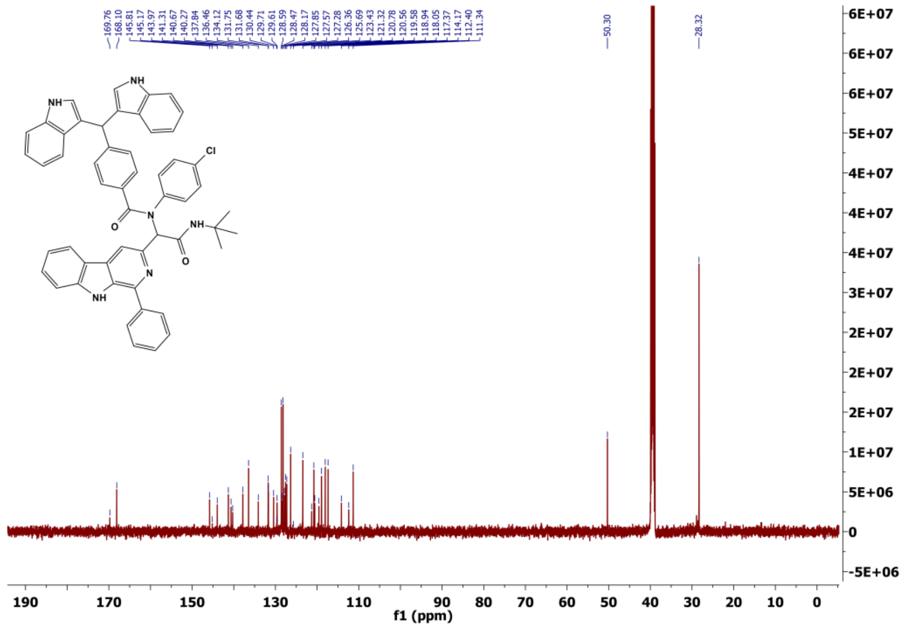

S21. Mass Spectra of Compound 12d

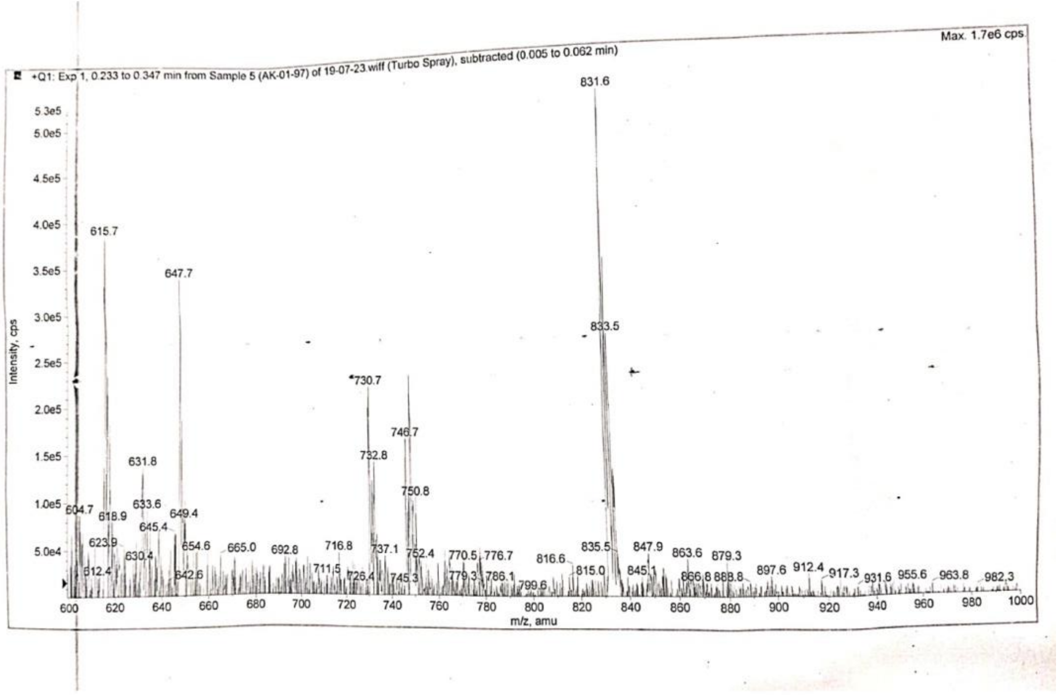

S22. FT-IR Spectra of Compound 12d

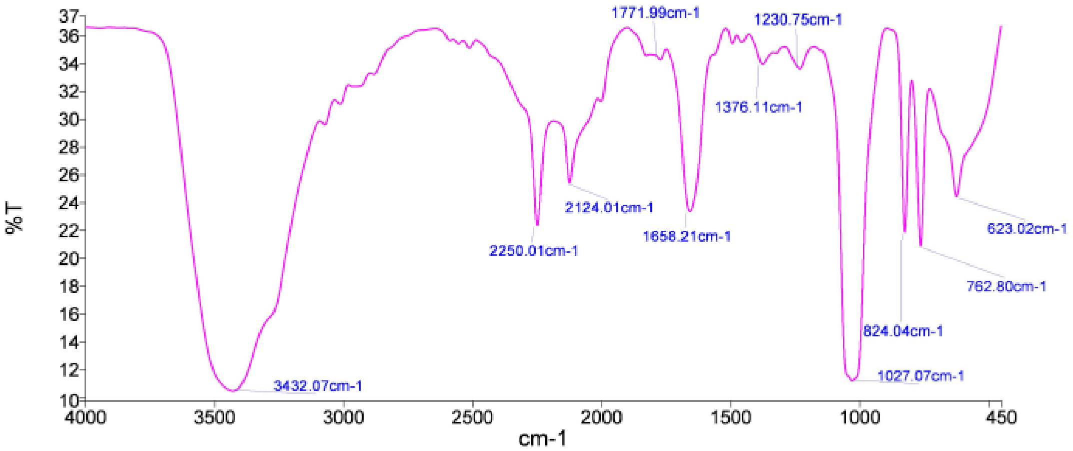

S23. ^{1}H NMR Spectra of Compound 12e in DMSO-d6

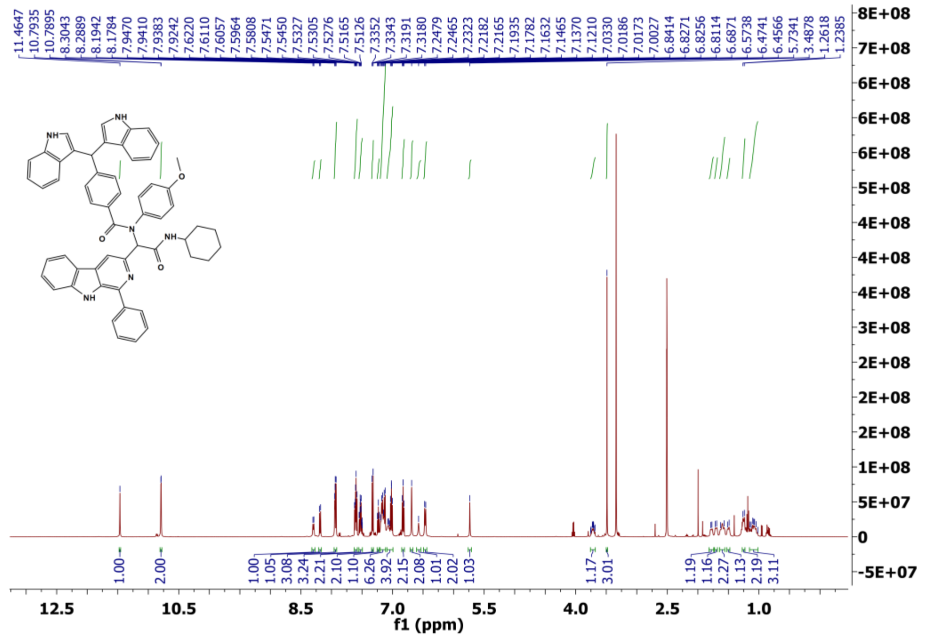

S24. ^{13}C NMR Spectra of Compound 12e in DMSO-d6

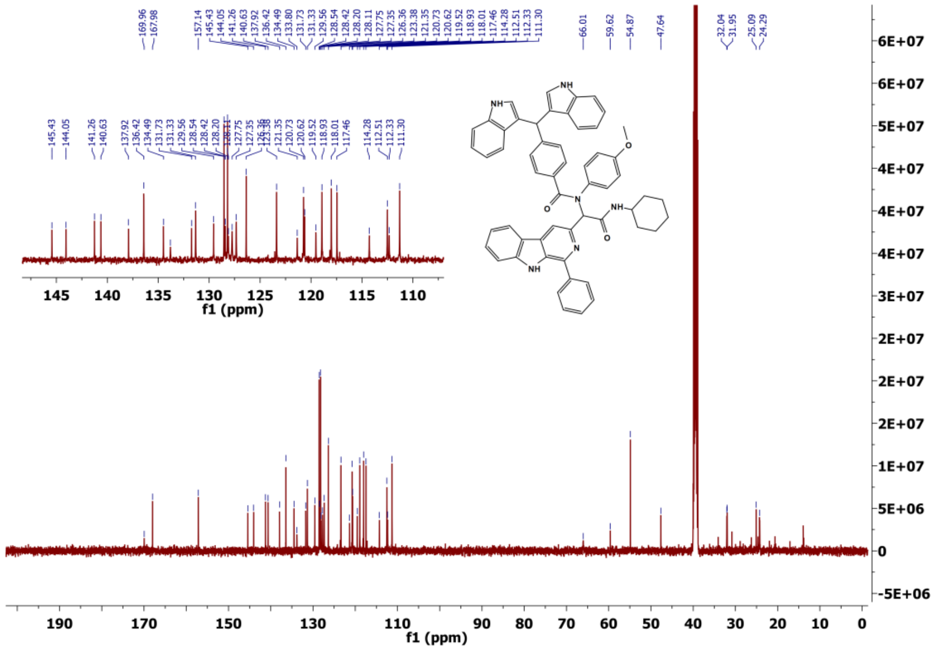

S25. Mass Spectra of Compound 12e

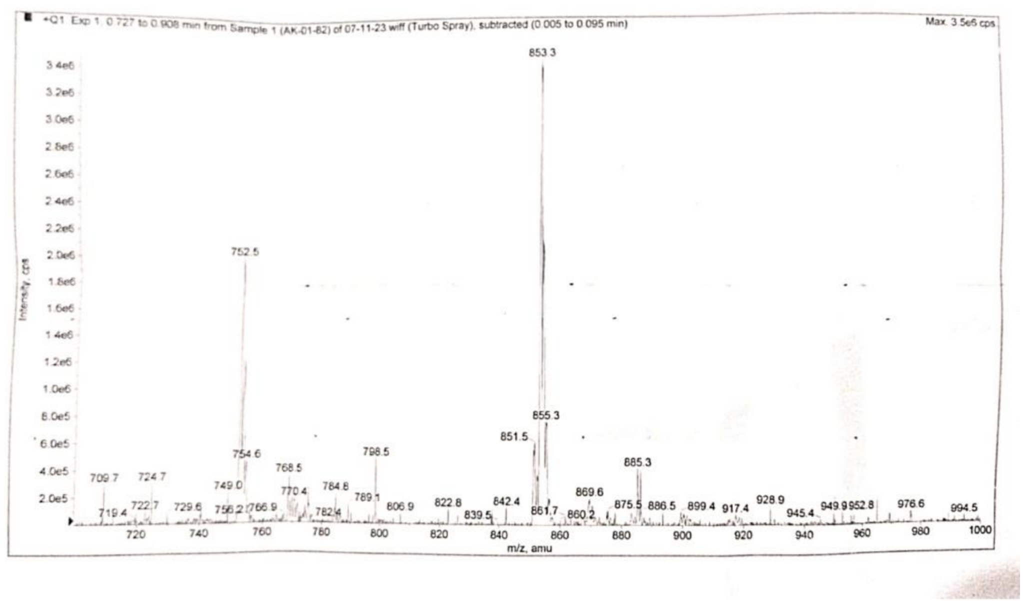

S26. FT-IR Spectra of Compound 12e

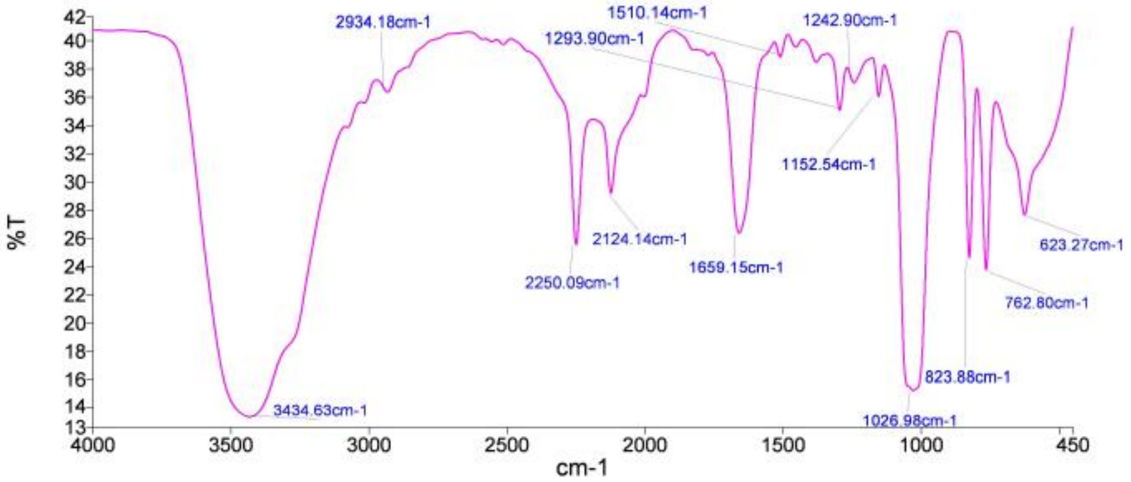

S27. ^{1}H NMR Spectra of Compound 12f in DMSO-d6

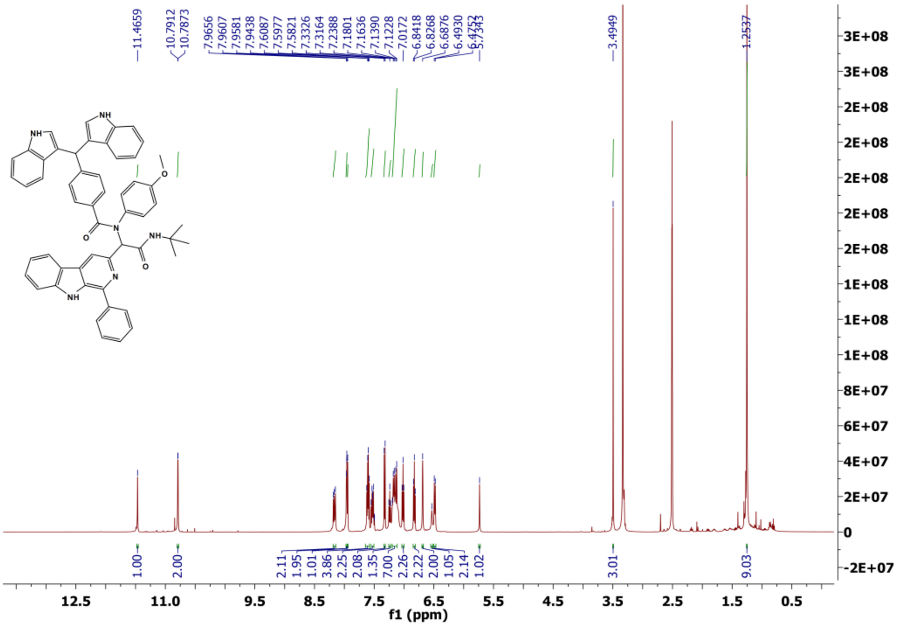

S28. ^{13}C NMR Spectra of Compound 12f in DMSO-d6

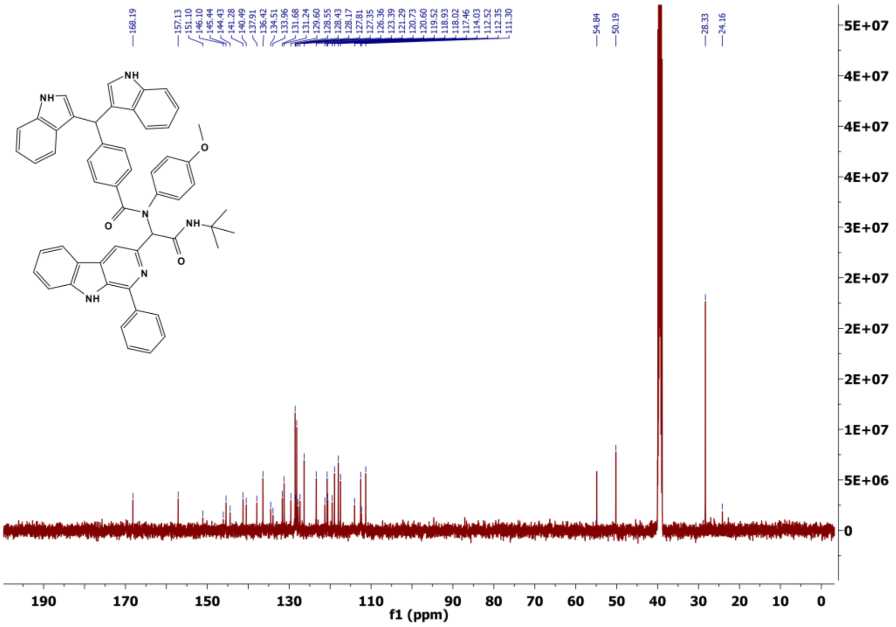

S29. Mass Spectra of Compound 12f

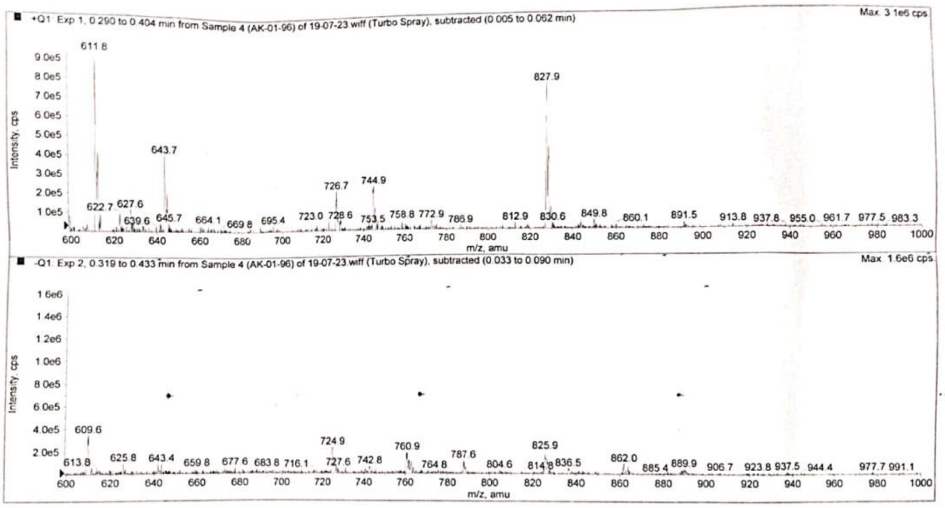

S30. FT-IR Spectra of Compound 12f

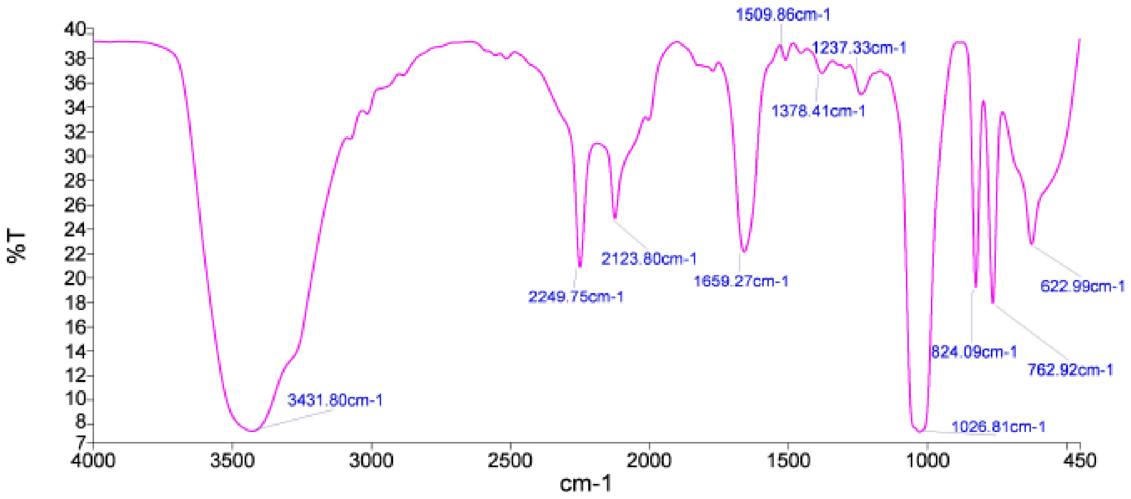

S31. ^{1}H NMR Spectra of Compound 12g in DMSO-d6

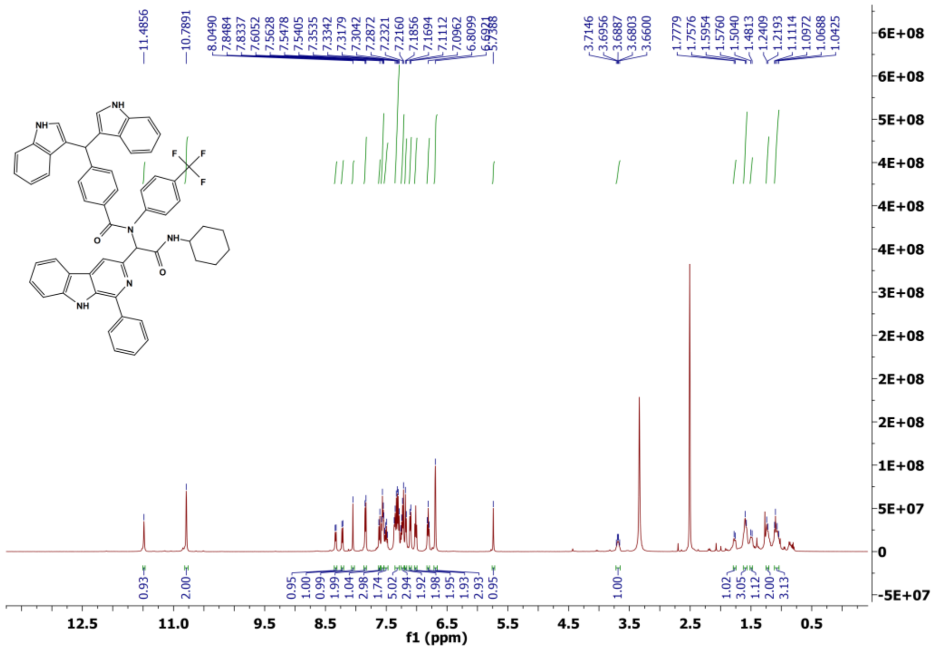

S32. ^{13}C NMR Spectra of Compound 12g in DMSO-d6

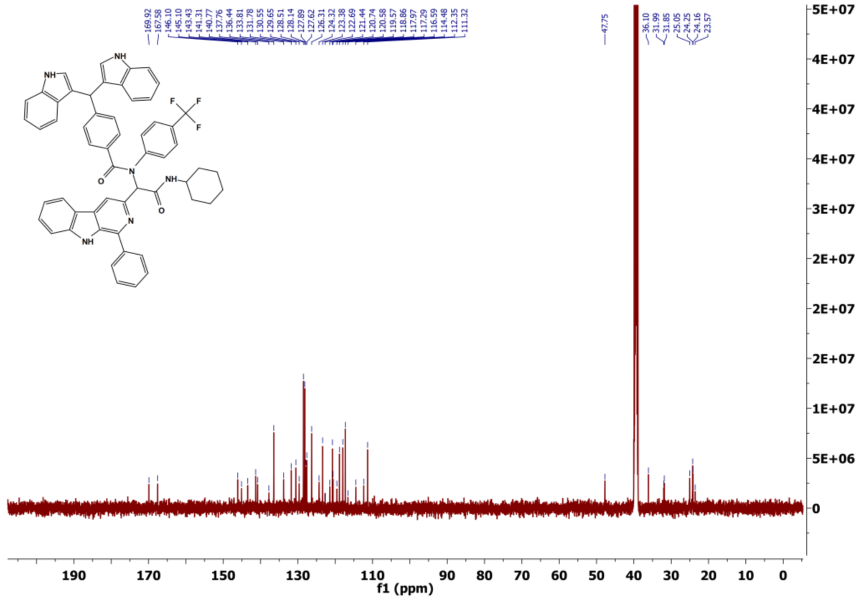

S33. Mass Spectra of Compound 12g

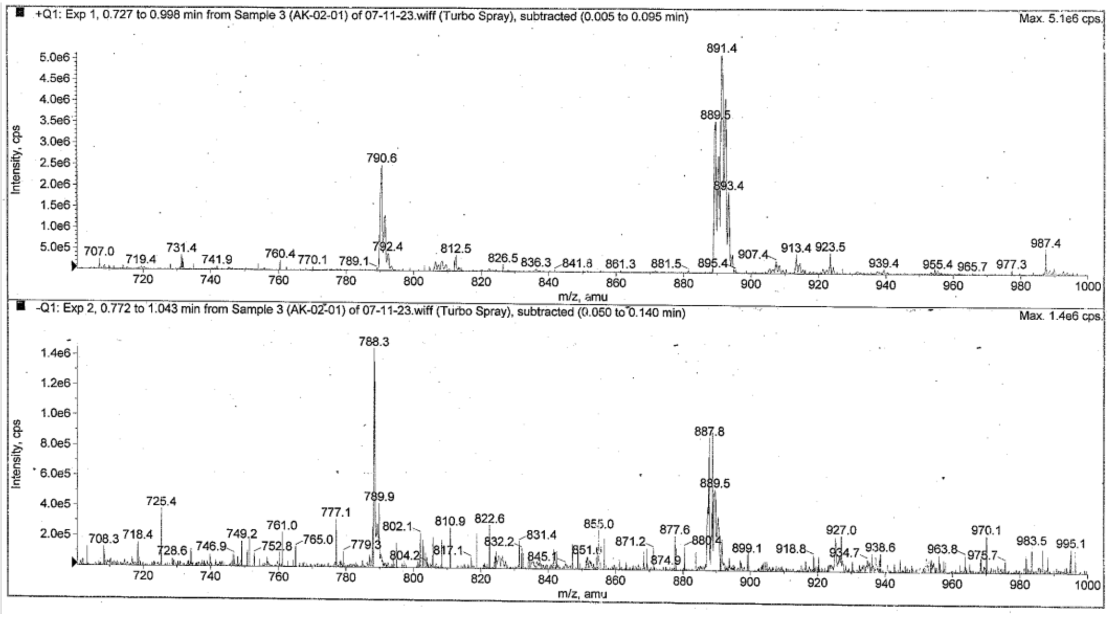

S34. FT-IR Spectra of Compound 12g

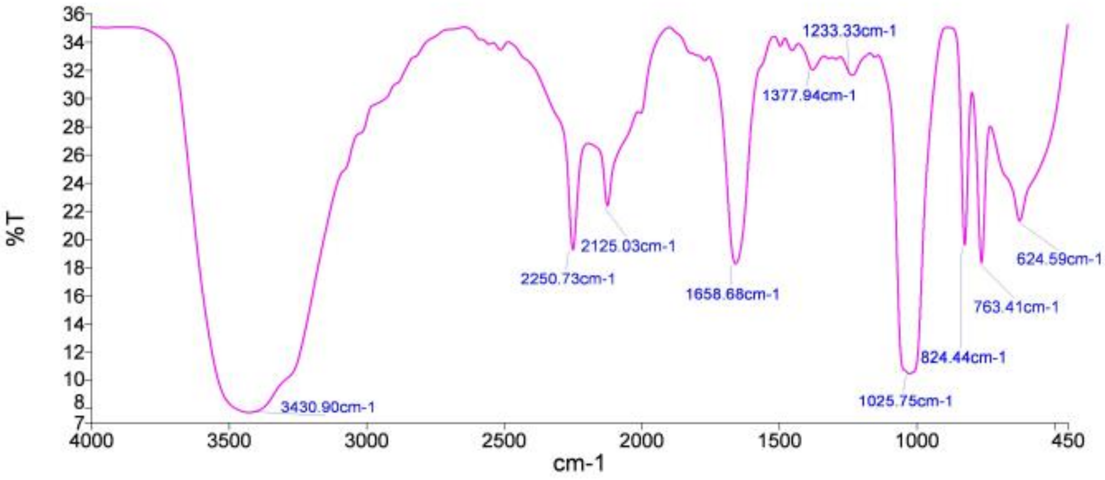

S35. ^{1}H NMR Spectra of Compound 12h in DMSO-d6

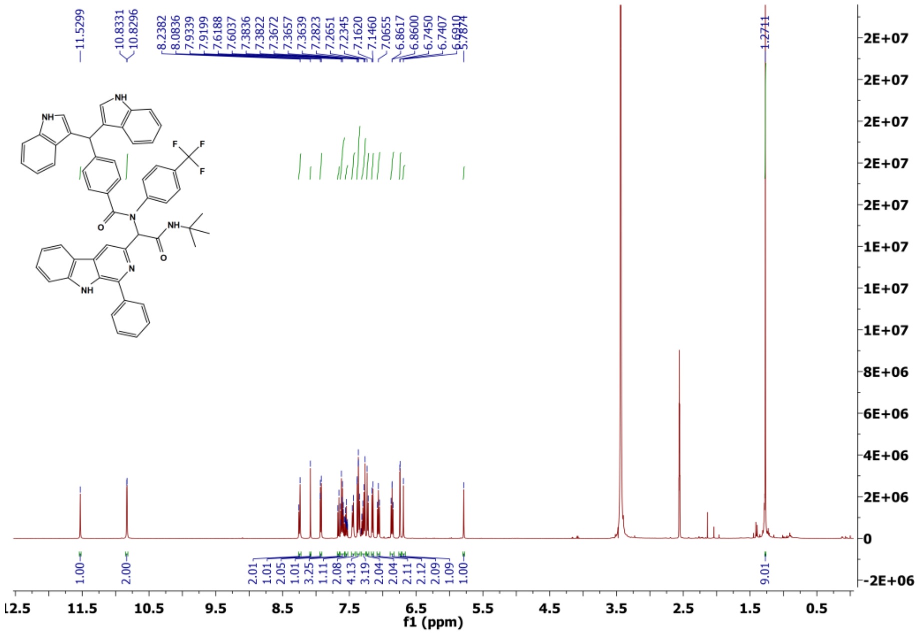

S36. ^{13}C NMR Spectra of Compound 12h in DMSO-d6

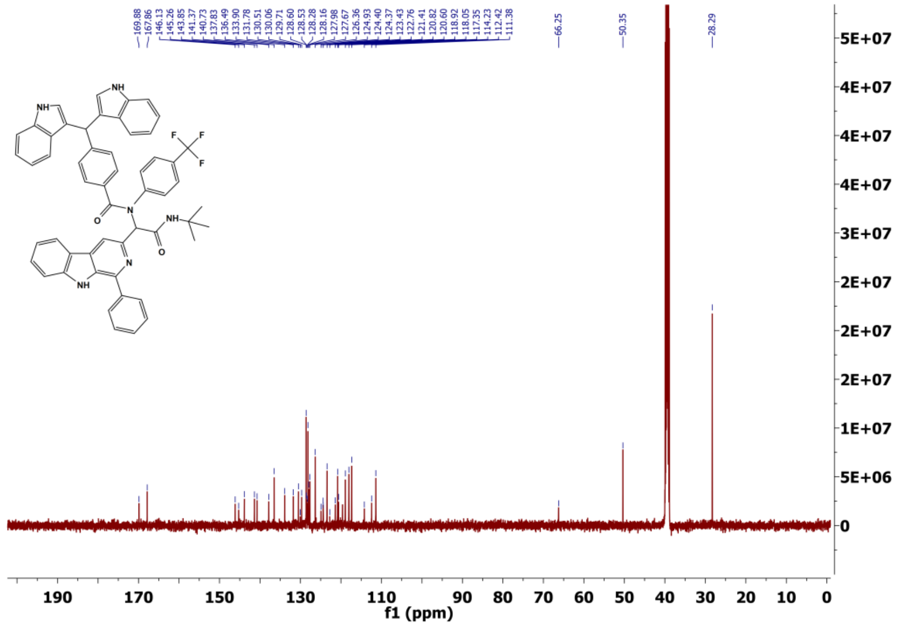

S37. Mass spectra of Compound 12h

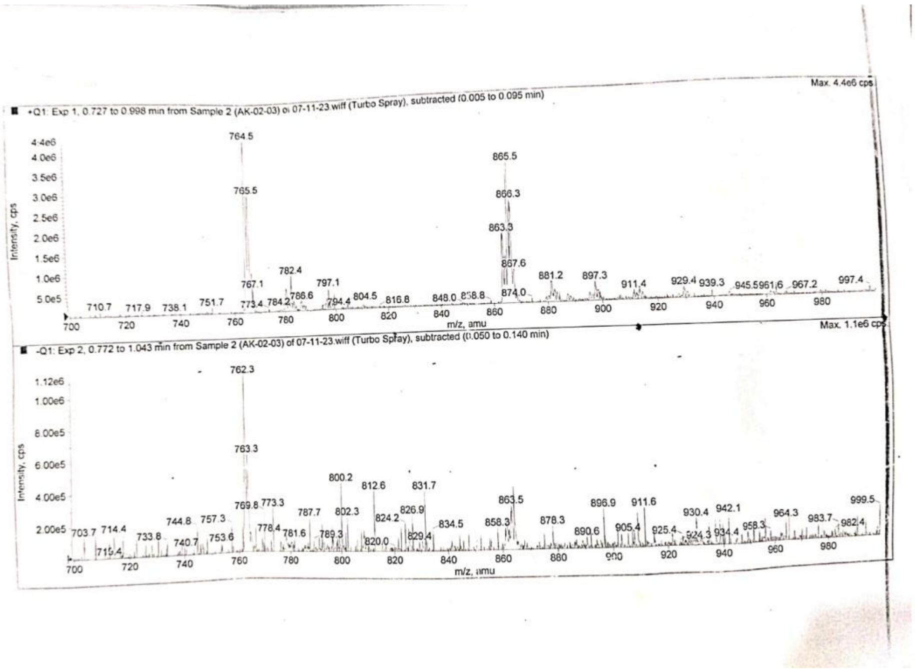

S38. FT-IR Spectra of Compound 12h

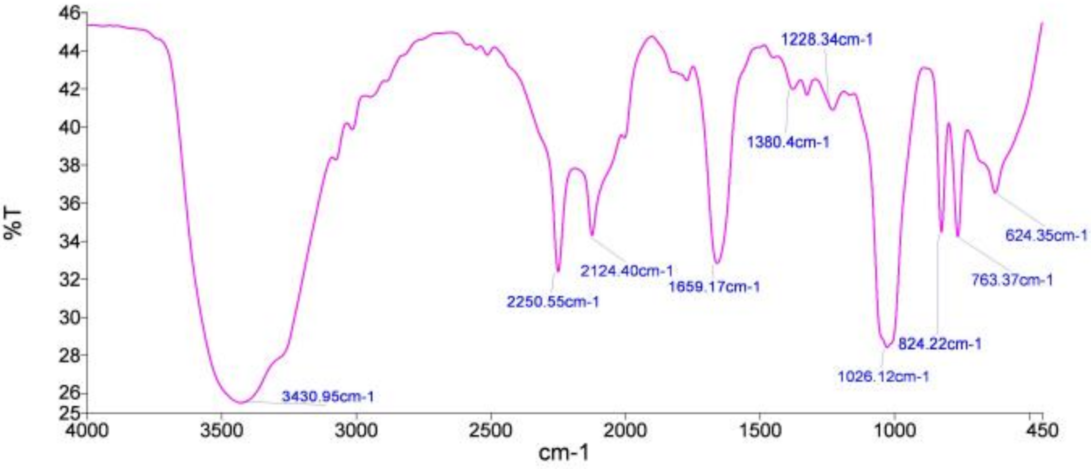

S39. ^{1}H NMR Spectra of Compound 12i in DMSO-d6

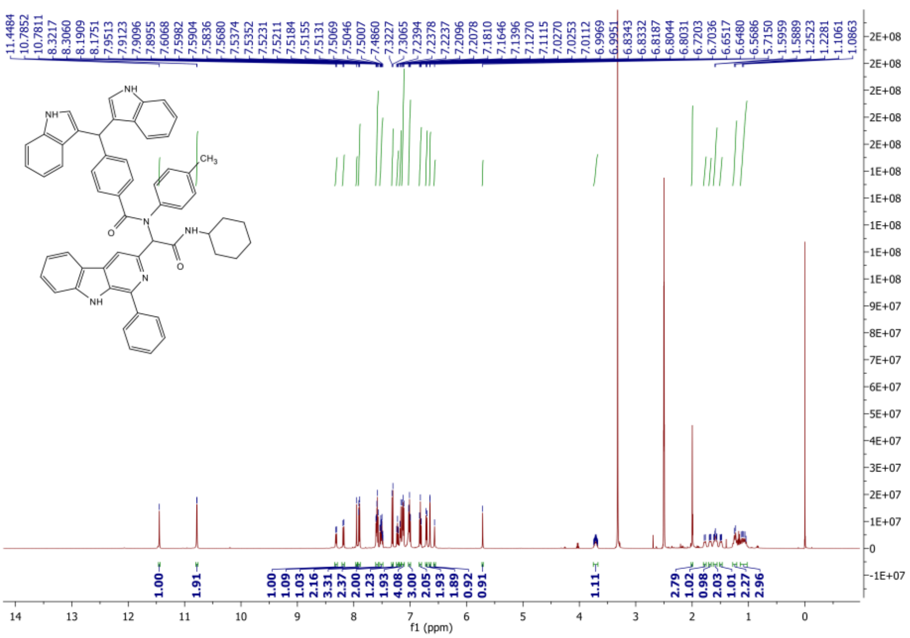

S40. ^{13}C NMR Spectra of Compound 12i in DMSO-d6

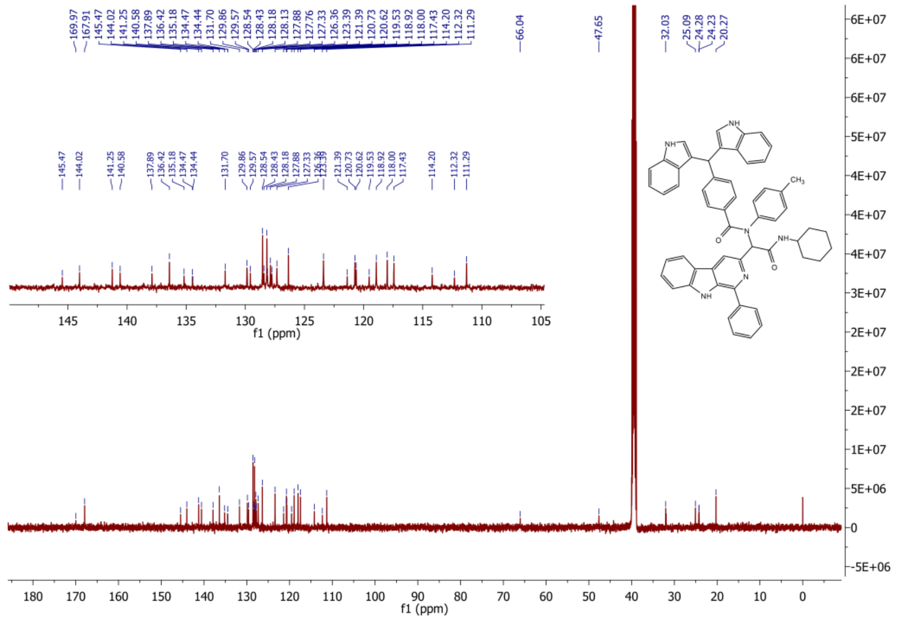

S41. Mass spectra of Compound 12i

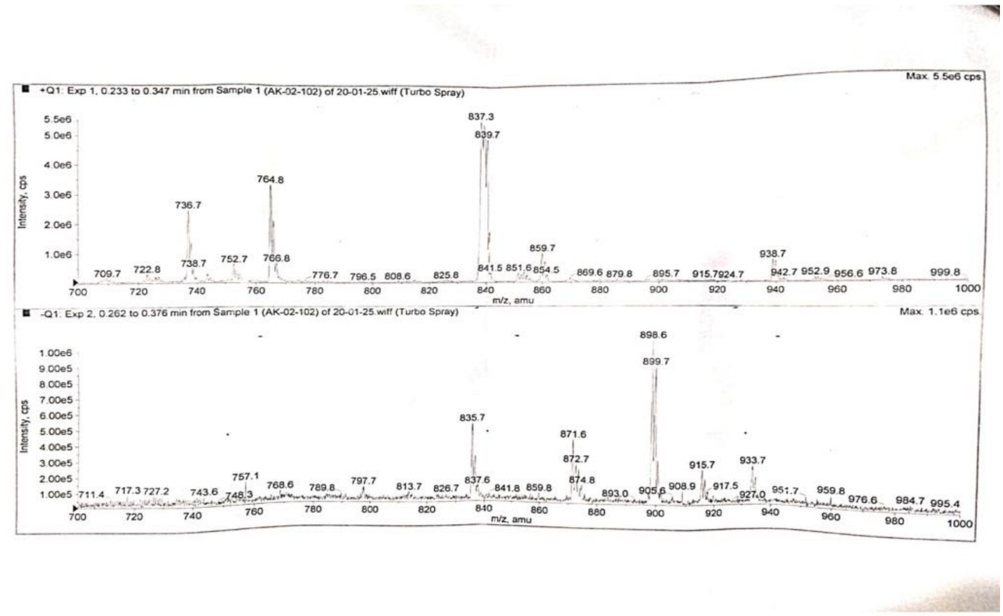

S42. FT-IR Spectra of Compound 12i

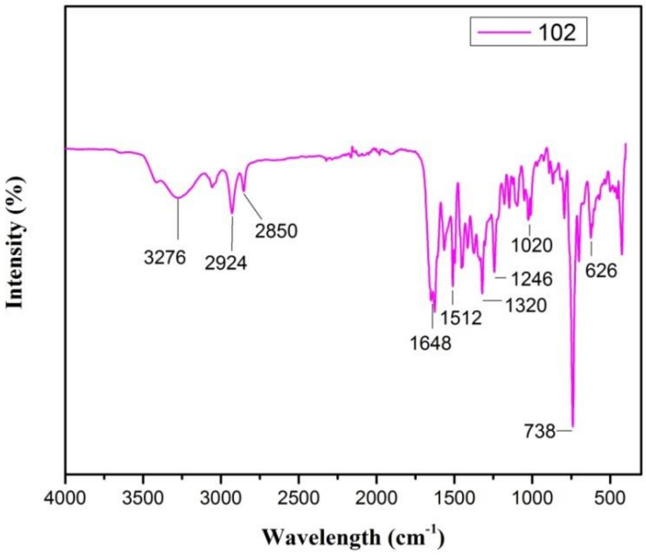

S43. ^{1}H NMR Spectra of Compound 12j in $CDCl_3$

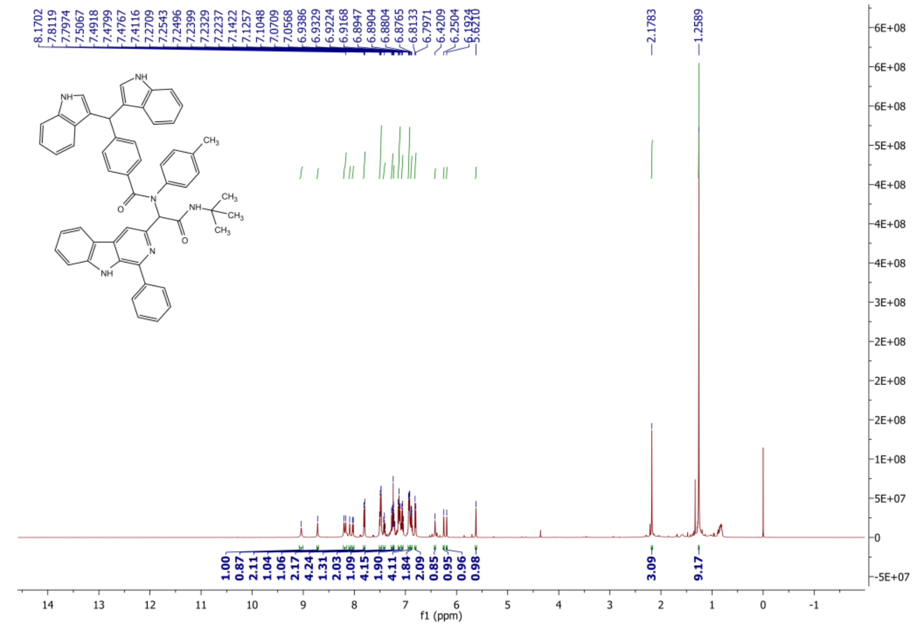

S44. ^{13}C NMR Spectra of Compound 12j in $CDCl_3$

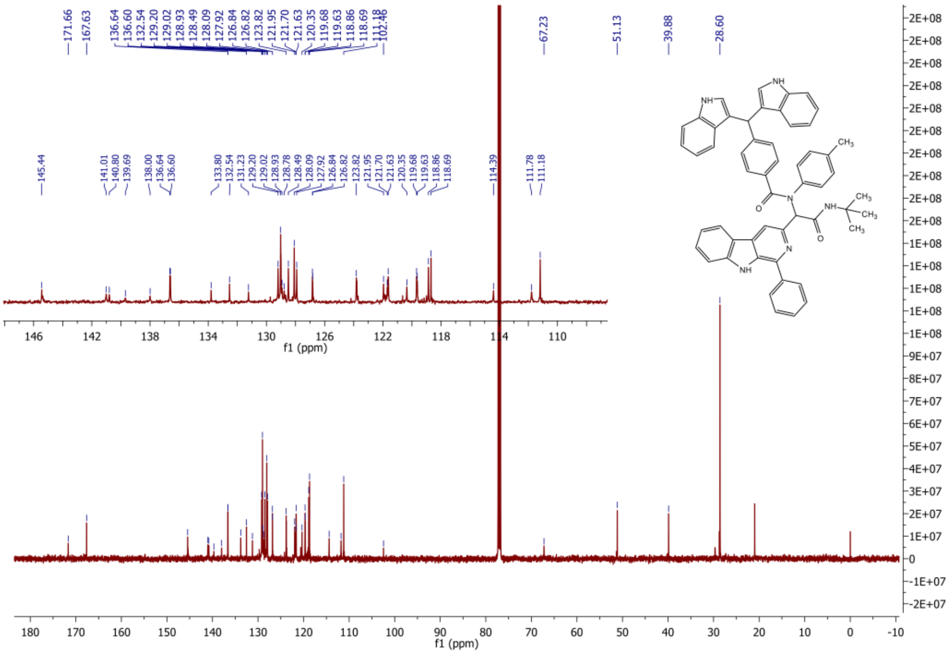

S45. Mass spectra of Compound 12j

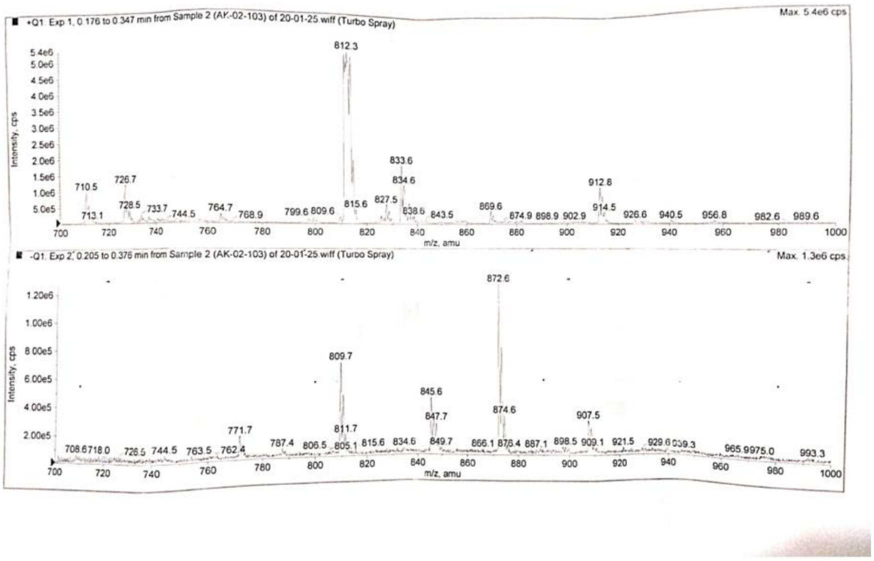

S46. FT-IR Spectra of Compound 12j

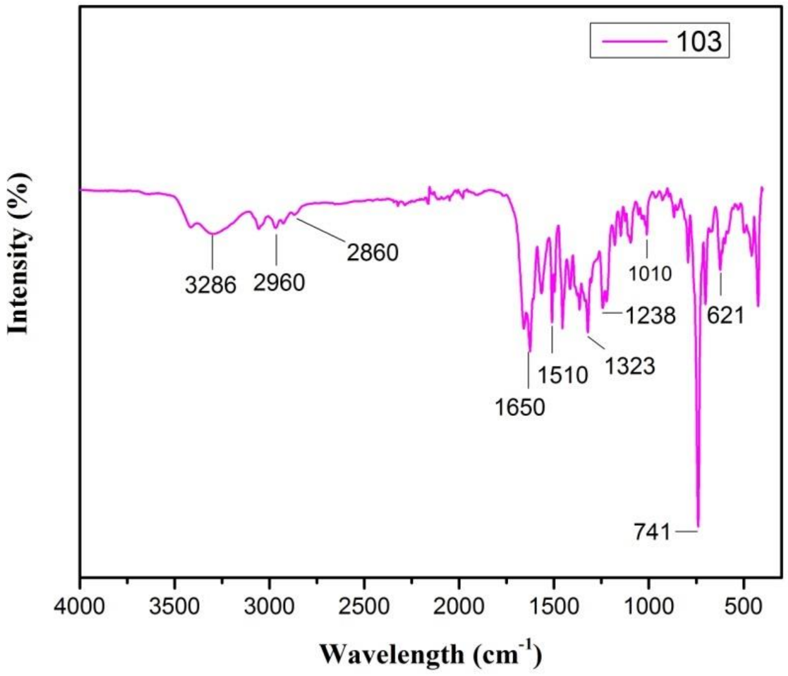

S47. ^{1}H NMR Spectra of Compound 12k in DMSO-d6

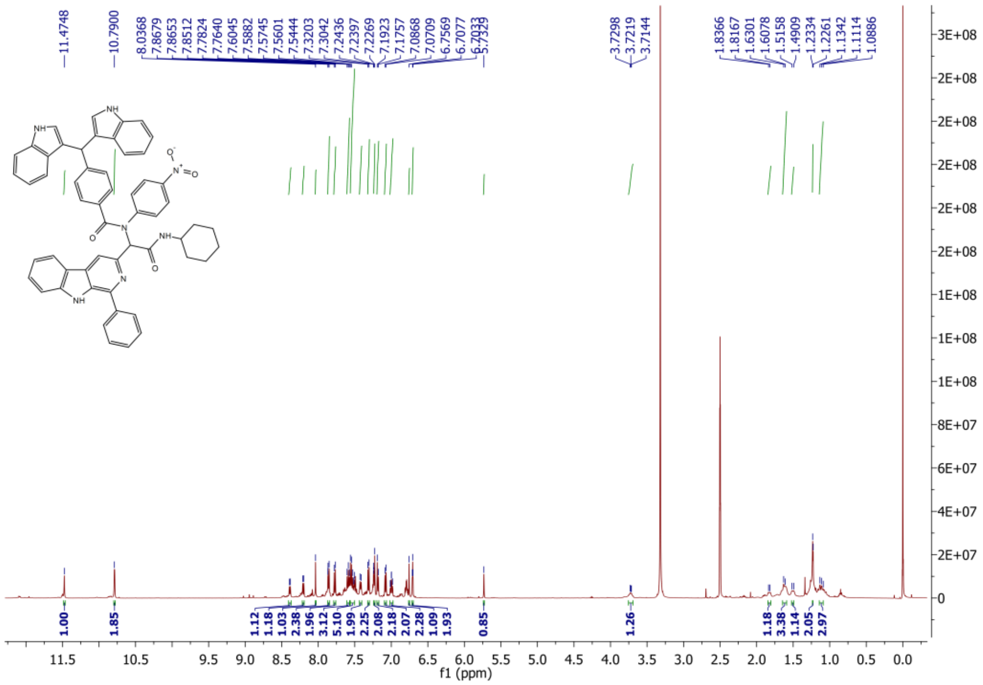

S48. ^{13}C NMR Spectra of Compound 12k in DMSO-d6

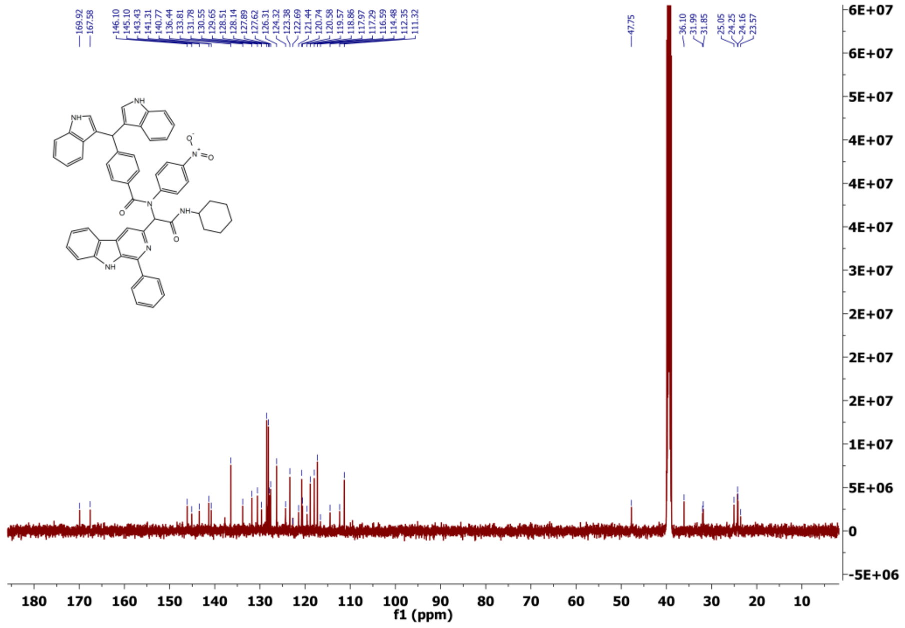

S49. Mass spectra of Compound 12k

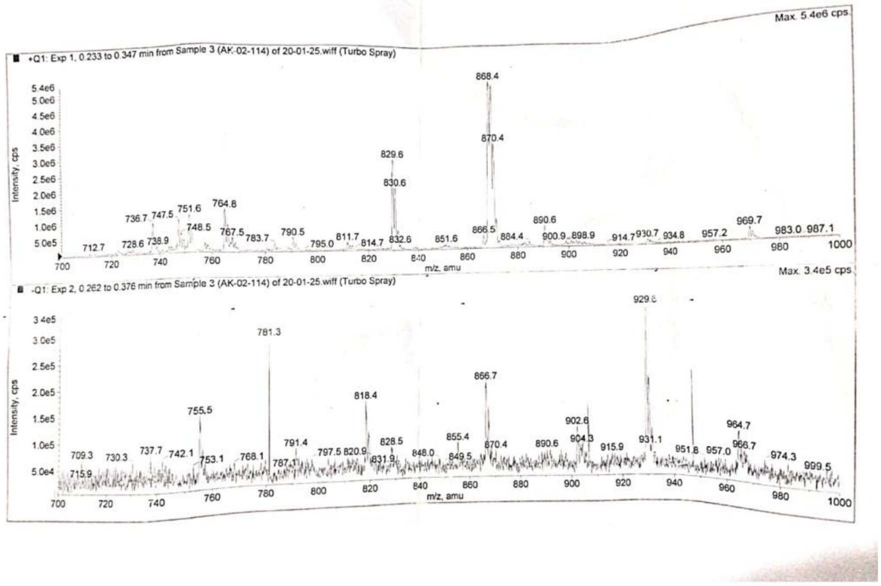

S50. FT-IR Spectra of Compound 12k

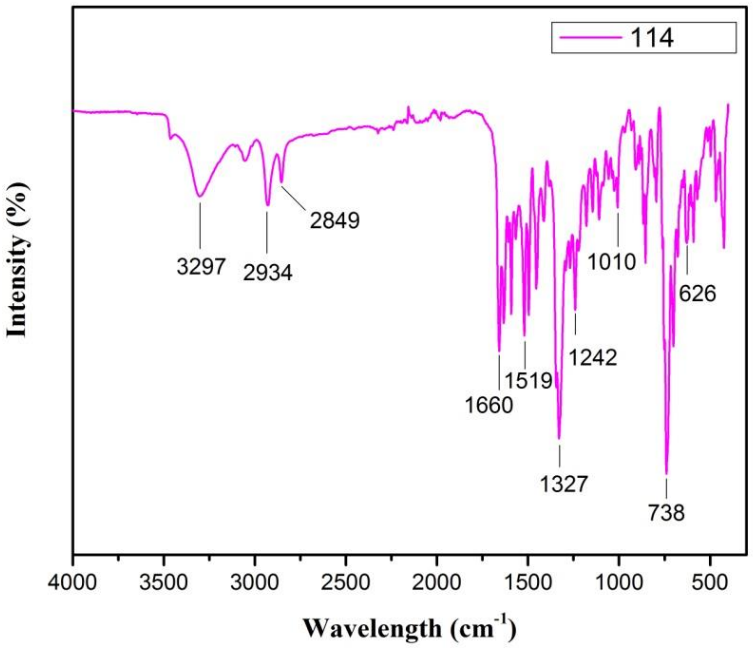

S51. ^{1}H NMR Spectra of Compound 12l in $CDCl_3$

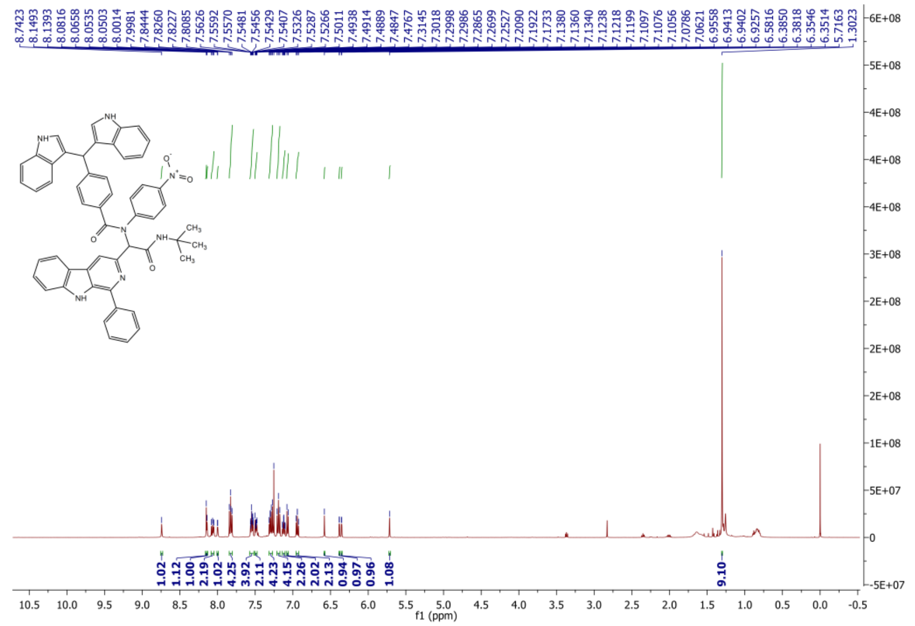

S52. ^{13}C NMR Spectra of Compound 12l in $CDCl_3$

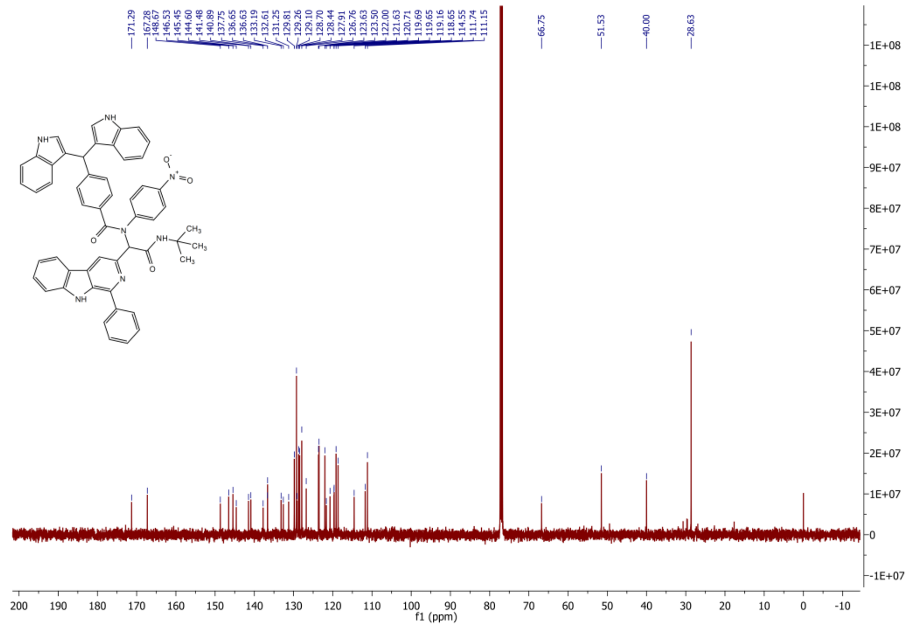

S53. Mass spectra of Compound 12l

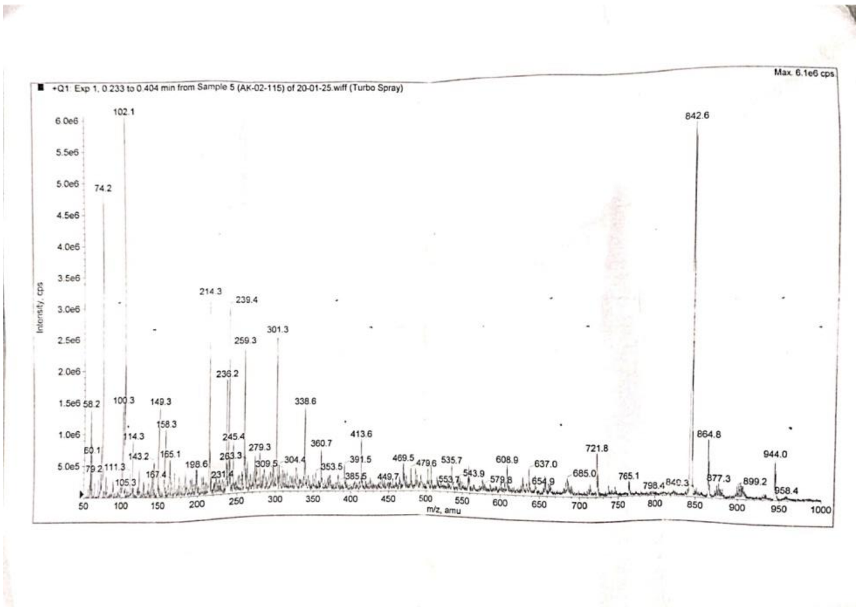

S54. FT-IR Spectra of Compound 12l

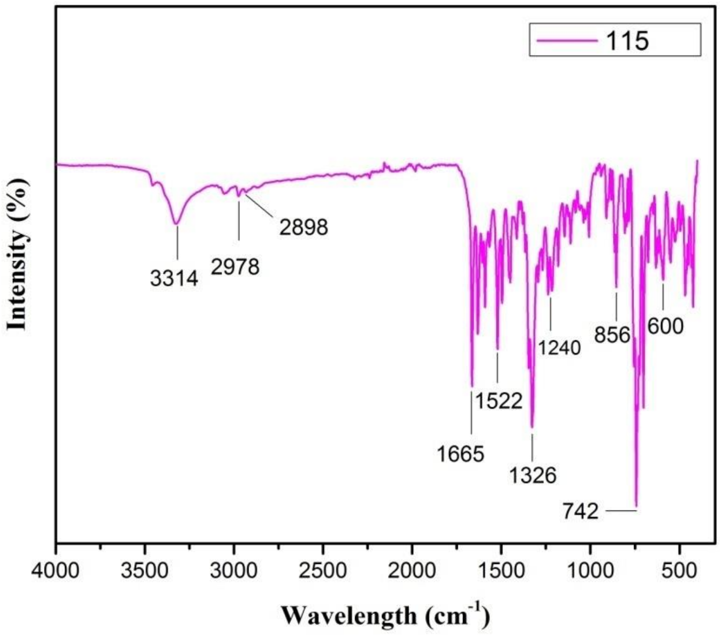

2. BIOLOGY

A. Evaluating the antibacterial potential of hybrids against Gram-negative and Gram-positive bacteria

To assess the in vitro antibacterial activity of the synthesized hybrid compounds, agar-diffusion assay was employed using Kirby-Bauer method against gram positive and gram negative bacteria. Two gram positive strains *Staphylococcus aureus, Bacillus subtilis* and two Gram negative strains *Pseudomonas*

aeruginosa, Acinetobacter baumannii were used to screen the antibacterial activity of the hybrids. Briefly, 100 µl of the bacterial cultures in their active phase of growth (containing 1 x 10^8 CFU/mL) were swabbed onto Mueller Hinton (MH) agar plates. Subsequently, the freshly seeded plates were punched with five wells each of 8 mm diameter using sterile borer. First two wells were then filled with 100 µl of reference/parent compounds at concentration of 5 mg/mL. To the third well, 100 µl of hybrid compound (5 mg/mL) was added. 100 µl of 50 µg/mL gentamicin (standard drug) and 100 µl of DMSO (solvent control) was added to the 4th and 5th well respectively. The agar plates were then incubated at 37 °C for 18 h and the zones of inhibition (in mm) were measured the next day. To achieve results that were statistically significant, each hybrid was examined three times.

B. Determining the minimum inhibitory concentrations (MIC) of the hybrids

Previously established protocols were used to determine the MIC of the synthesized hybrids for the selected Gram positive and Gram-negative bacterial strains using standard 96 well microtiter broth dilution method. In each well, 100 µl of double-strength MH broth was added followed by dispensing 100 µl of hybrid compound (5 mg/mL) in the first well. The compounds were then serially diluted (1:1) by transferring 100 µl suspension from previous well to the next one, repeating this process until reaching the desired lowest dilutions of the compounds. To each well, barring the negative controls, 10 µl of bacterial inoculum was added. Negative control wells consisted of only MH broth while positive growth controls included wells with inoculated MH broth devoid of hybrid compounds. The MIC plates were incubated at 37 °C for 18-20 h. Next day, 20 µl of resazurin dye (working concentration 0.01%) was added to each well followed by incubation at 37 °C for 2 h. Since resazurin is a redox dye, a visible colour change from blue to pink was observed in the wells with microbial growth. However, the lowest concentration at which there was no visible colour change after the addition of the dye was identified as the MIC, indicating complete inhibition of microbial growth.

S55. In vitro antibacterial activity of the synthesized compounds 12 a-l

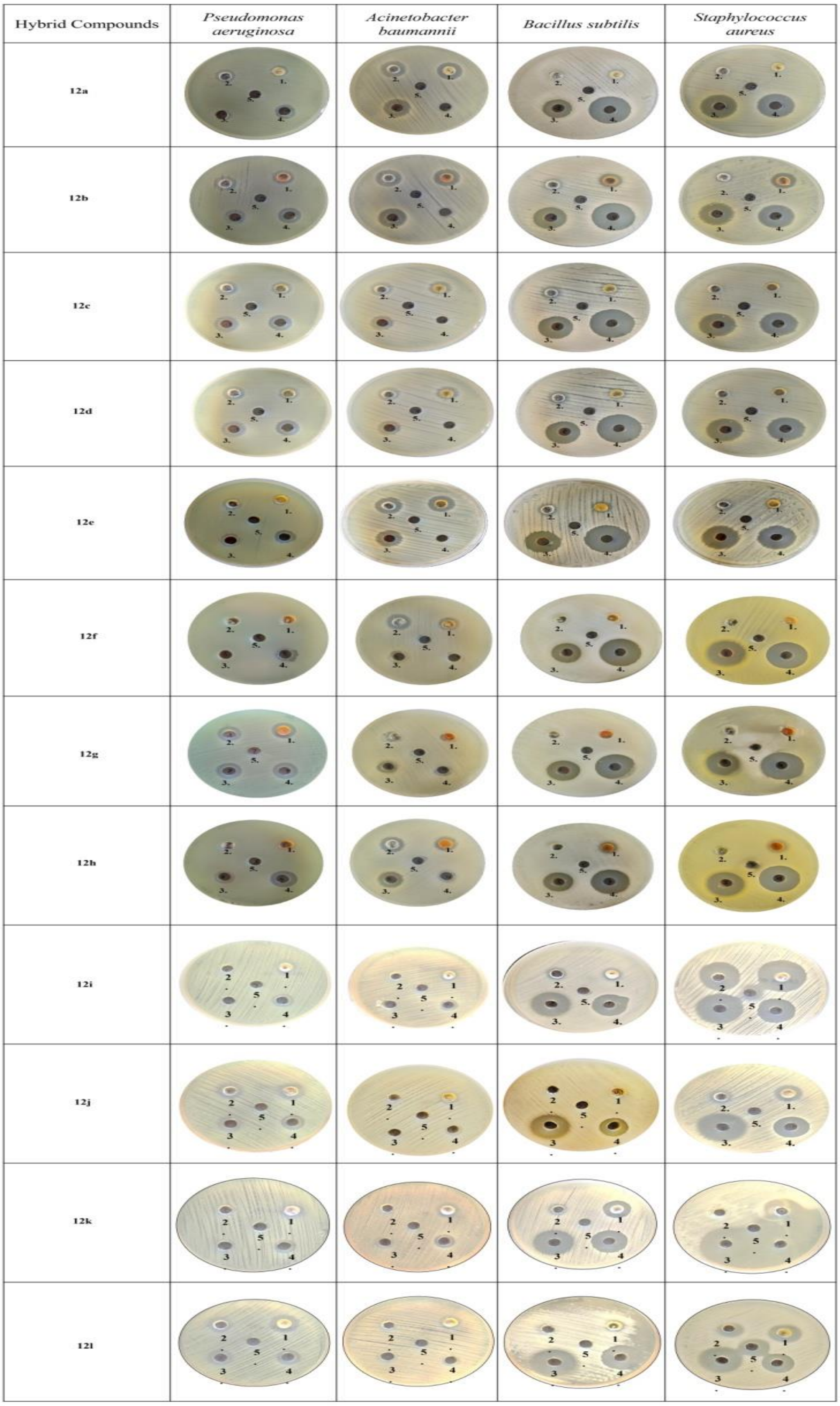

3. Molecular docking

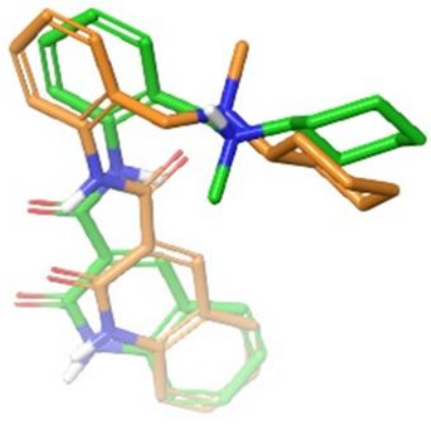

Figure S56. Docking protocol validation. Alignment of the ligand (orange colored, with PDB id: 6KZV) with the best-docked pose and its crystallographic binding mode (in green).
